# Supplementary material for: Generation of hypoimmunogenic induced pluripotent stem cells by CRISPR-Cas9 system and detailed evaluation for clinical application
Source: Mol Ther Methods Clin Dev. 2022 May 29;26:15–25. doi: 10.1016/j.omtm.2022.05.010 (PMC9198376; doi:10.1016/j.omtm.2022.05.010)
Supplement: Document S1. Figures S1–S7 and Tables S1–S6 [file mmc1.pdf]

OMTM, Volume *26*

Supplemental information

Generation of hypoimmunogenic induced pluripotent stem cells by CRISPR-Cas9 system and detailed evaluation for clinical application

Yuko Kitano, Sayaka Nishimura, Tomoaki M. Kato, Anna Ueda, Kaho Takigawa, Masafumi Umekage, Masaki Nomura, Ayane Kawakami, Haruna Ogawa, Huaigeng Xu, Akitsu Hotta, Naoko Takasu, and Masayoshi Tsukahara

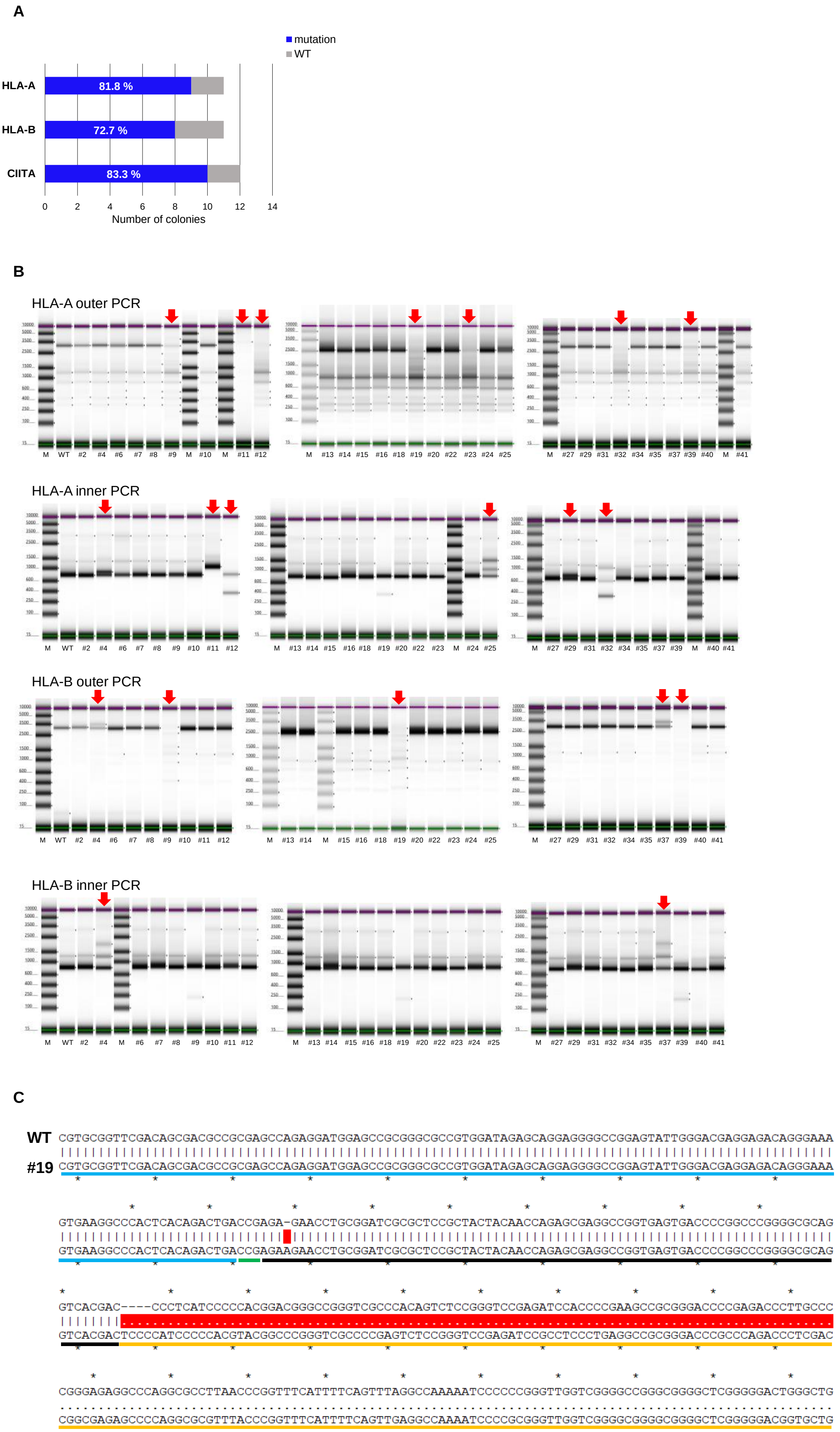
A
mutation
WT
HLA-A
81.8 %
HLA-B
72.7 %
CIITA
83.3 %
0 2 4 6 8 10 12 14
Number of colonies
B
HLA-A outer PCR
M WT #2 #4 #6 #7 #8 #9 M #10 M #11 #12
M #13 #14 #15 #16 #18 #19 #20 #22 #23 #24 #25
M #27 #29 #31 #32 #34 #35 #37 #39 #40 M #41
HLA-A inner PCR
M WT #2 #4 #6 #7 #8 #9 #10 #11 #12
M #13 #14 #15 #16 #18 #19 #20 #22 #23 M #24 #25
M #27 #29 #31 #32 #34 #35 #37 #39 M #40 #41
HLA-B outer PCR
M WT #2 #4 #6 #7 #8 #9 #10 #11 #12
M #13 #14 M #15 #16 #18 #19 #20 #22 #23 #24 #25
M #27 #29 #31 #32 #34 #35 #37 #39 #40 #41
HLA-B inner PCR
M WT #2 #4 M #6 #7 #8 #9 #10 #11 #12
M #13 #14 #15 #16 #18 #19 #20 #22 #23 #24 #25
M #27 #29 #31 #32 #34 #35 #37 #39 #40 #41
C
WT CGTGCGGTTCGACAGCGACGCCGCGAGCCAGAGGATGGAGCCGCGGGCGCCGTGGATAGAGCAGGAGGGGCCGGAGTATTGGGACGAGGAGACAGGGAAA
#19 CGTGCGGTTCGACAGCGACGCCGCGAGCCAGAGGATGGAGCCGCGGGCGCCGTGGATAGAGCAGGAGGGGCCGGAGTATTGGGACGAGGAGACAGGGAAA
GTGAAGGCCCACTCACAGACTGACCGAGA-GAACCTGCGGATCGCGCTCCGCTACTACAACCAGAGCGAGGCCGGTGAGTGACCCCGGCCCGGGGCGCAG
GTGAAGGCCCACTCACAGACTGACCGAGAAGAACCTGCGGATCGCGCTCCGCTACTACAACCAGAGCGAGGCCGGTGAGTGACCCCGGCCCGGGGCGCAG
GTCACGAC----CCCTCATCCCCCACGGACGGGCCGGGTCGCCCACAGTCTCCGGGTCCGAGATCCACCCCGAAGCCGCGGGACCCCGAGACCCTTGCCC
GTCACGACTCCCCATCCCCCACGTACGGCCCGGGTCGCCCCGAGTCTCCGGGTCCGAGATCCGCCTCCCTGAGGCCGCGGGACCCGCCCAGACCCTCGAC
CGGGAGAGGCCCAGGCGCCTTAACCCGGTTTCATTTTCAGTTTAGGCCAAAAATCCCCCCGGGTTGGTCGGGGCCGGGCGGGGCTCGGGGGACTGGGCTG
CGGCGAGAGCCCCAGGCGCGTTTACCCGGTTTCATTTTCAGTTGAGGCCAAAATCCCCGCGGGTTGGTCGGGGCGGGGCGGGGCTCGGGGGACGGTGCTG

D

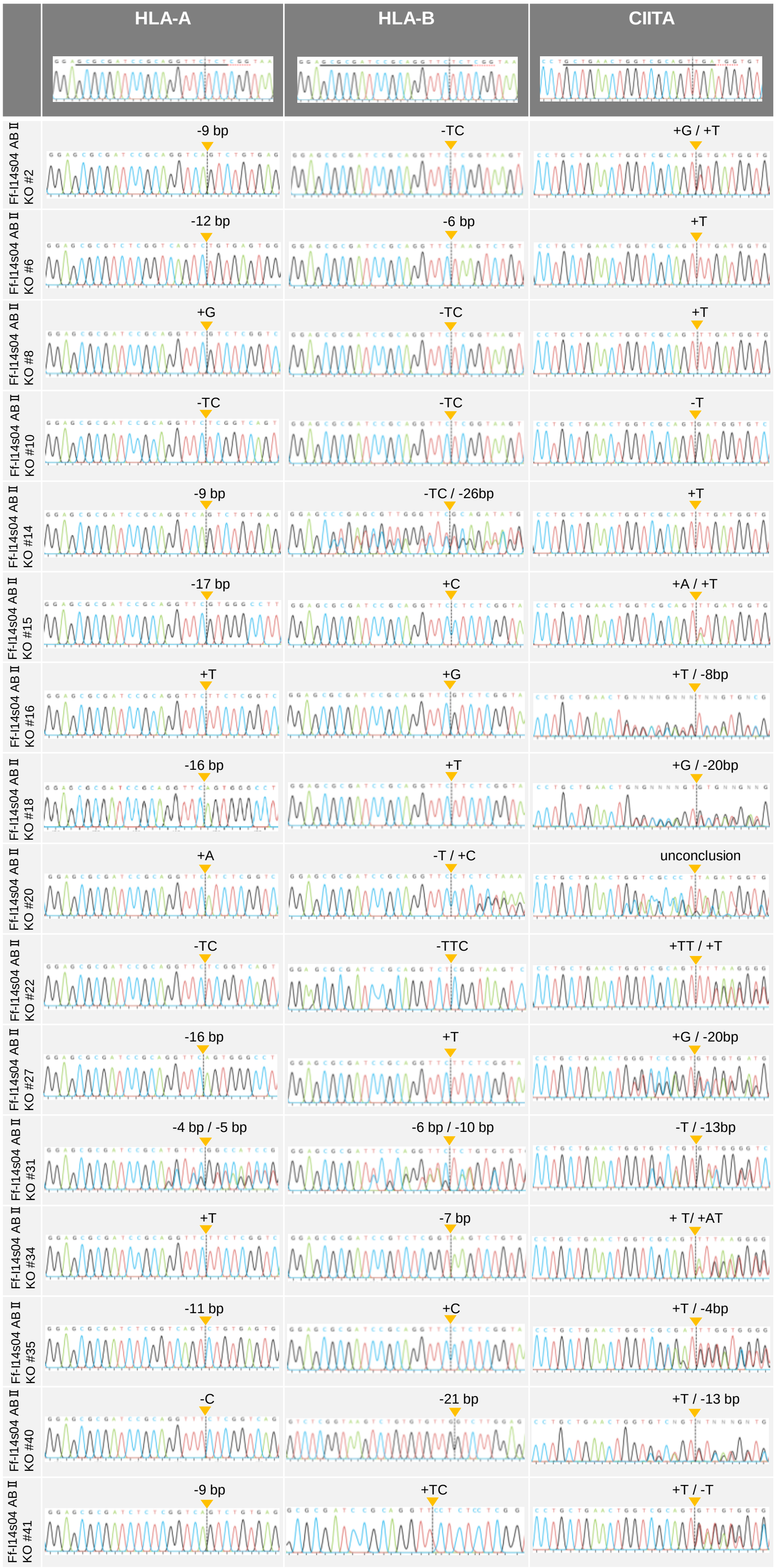

HLA-A
HLA-B
CIITA
Ff-I14s04 AB II KO #2
-9 bp
-TC
+G / +T
Ff-I14s04 AB II KO #6
-12 bp
-6 bp
+T
Ff-I14s04 AB II KO #8
+G
-TC
+T
Ff-I14s04 AB II KO #10
-TC
-TC
-T
Ff-I14s04 AB II KO #14
-9 bp
-TC / -26bp
+T
Ff-I14s04 AB II KO #15
-17 bp
+C
+A / +T
Ff-I14s04 AB II KO #16
+T
+G
+T / -8bp
Ff-I14s04 AB II KO #18
-16 bp
+T
+G / -20bp
Ff-I14s04 AB II KO #20
+A
-T / +C
unconclusion
Ff-I14s04 AB II KO #22
-TC
-TTC
+TT / +T
Ff-I14s04 AB II KO #27
-16 bp
+T
+G / -20bp
Ff-I14s04 AB II KO #31
-4 bp / -5 bp
-6 bp / -10 bp
-T / -13bp
Ff-I14s04 AB II KO #34
+T
-7 bp
+ T/ +AT
Ff-I14s04 AB II KO #35
-11 bp
+C
+T / -4bp
Ff-I14s04 AB II KO #40
-C
-21 bp
+T / -13 bp
Ff-I14s04 AB II KO #41
-9 bp
+TC
+T / -T

Figure S1. Analysis of the target site of HLA-A/HLA-B/CIITA genome-edited iPSCs.

A) Histogram showing the frequency of WT and mutation sequences in the bulk cell population transformed with each sgRNA construct. The horizontal axis indicates the number of colonies sequenced.

B) Automated electrophoresis of PCR amplified products using HLA-A outer/inner and HLA-B outer/inner specific PCR primer sets. Lanes #1-41 were examined genome-edited iPSC clones. Lane M, 10kb DNA size marker. Red arrows show the clones that were different size from WT or for which PCR products were not obtained.

C) Sequence alignment of Sanger reads of HLA-A from origin (WT) and Ff-I14s04 ABII KO #19. cyan: HLA-A sequence, green: PAM, black: HLA-A/HLA-B common sequence, yellow: HLA-B sequence.

D) Electropherograms of Sanger sequences at the target sites in genome-edited iPSC clones.

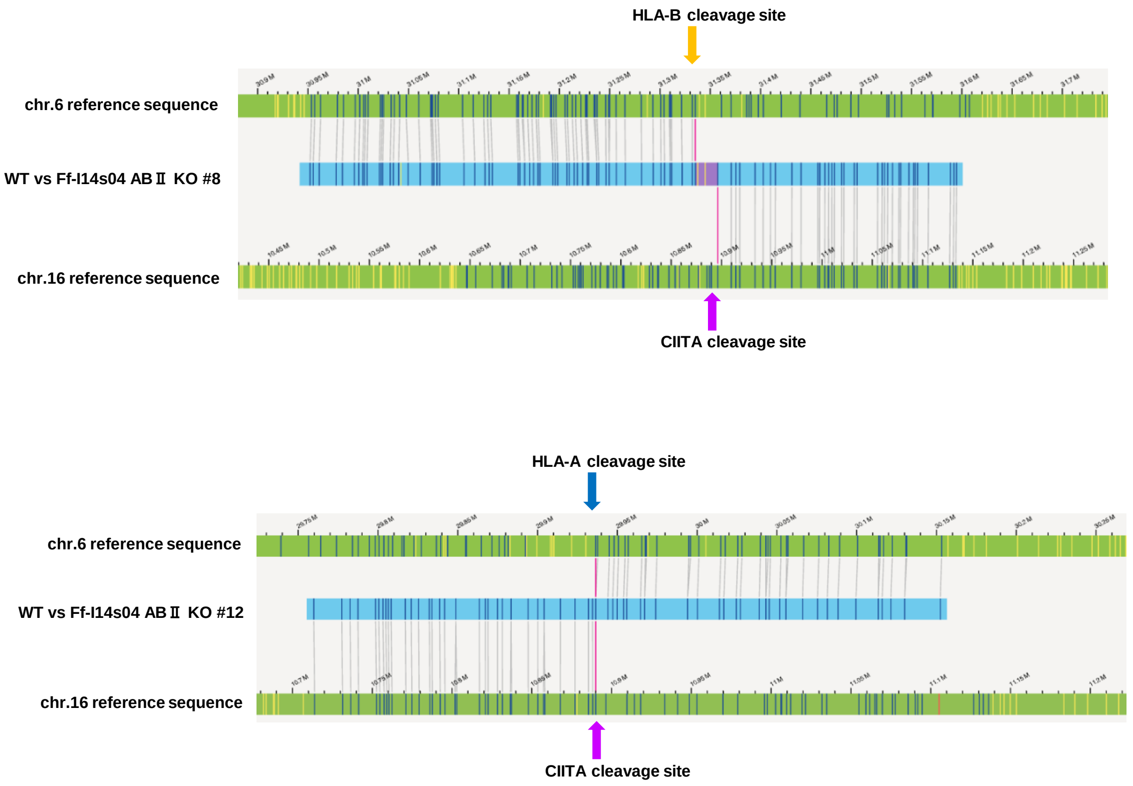

Figure S2. Complicated genome sequence shown by whole genome analysis.

The chromosomal translocations between HLA-A or HLA-B and CIITA cleavage site observed in optical genome mapping.

The genomic coordinate is hg38.

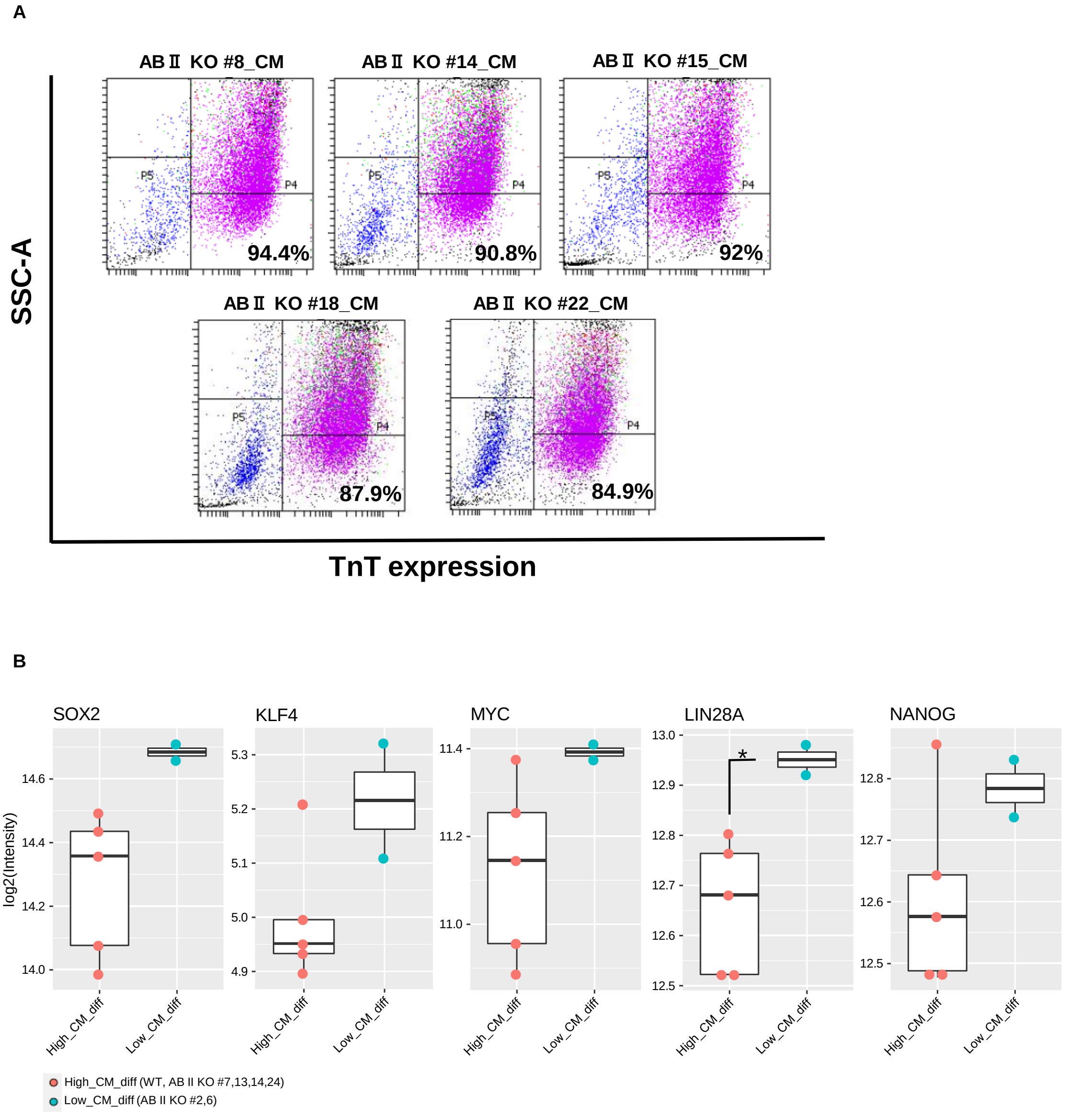

Figure S3. Evaluation of the cardiomyocyte differentiation potential of cells derived from genome-edited iPSCs.

A) Scatter plots of results of the FCM analysis examining the proportion of troponin T (TnT)+ cells after the induction of differentiation. The horizontal axis indicates the fluorescence intensity of fluorophores conjugated with the TnT antibody. The vertical axis indicates side scatter.

B) Boxplot of microarray data showing the expressions levels of pluripotent marker genes in iPSC clones showing high(orange) or low(cyan) cardiomyocyte differentiation potential. Each dot shows the clones. Asterisk indicates statistical significance: * $p < 0.05$.

A

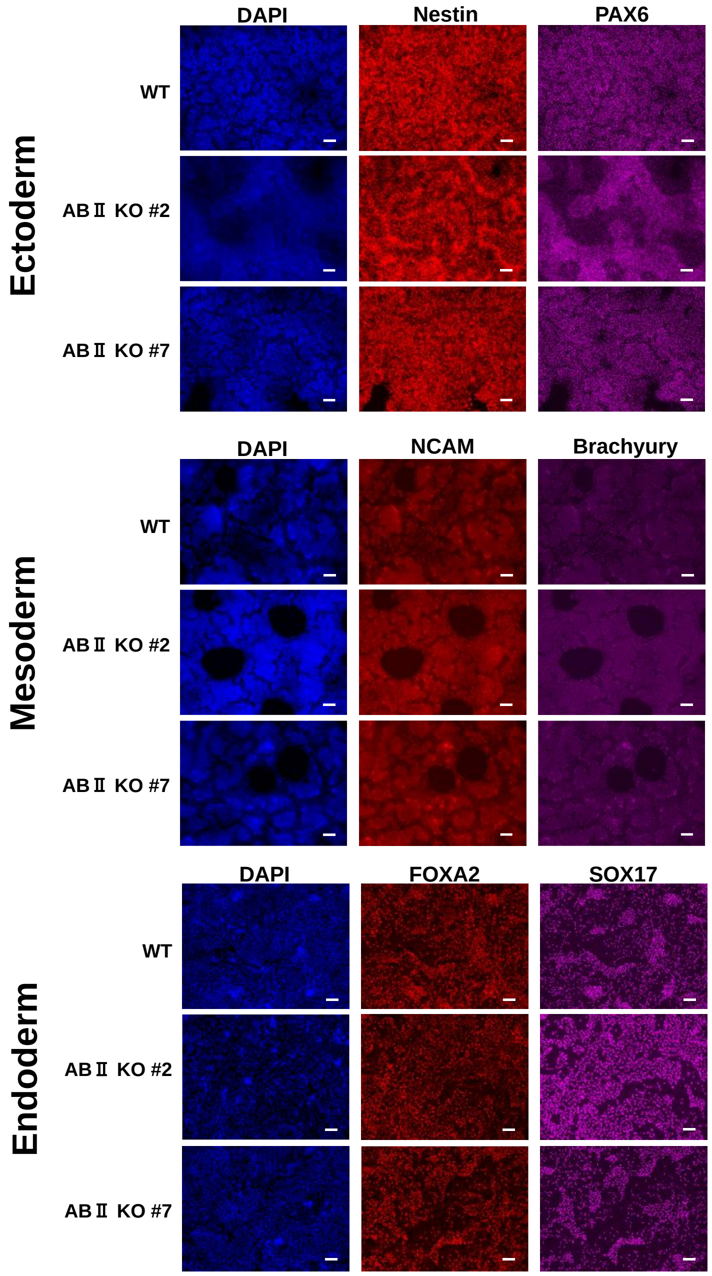
DAPI
Nestin
PAX6
WT
ABⅡ KO #2
ABⅡ KO #7
Ectoderm
DAPI
NCAM
Brachyury
WT
ABⅡ KO #2
ABⅡ KO #7
Mesoderm
DAPI
FOXA2
SOX17
WT
ABⅡ KO #2
ABⅡ KO #7
Endoderm

B

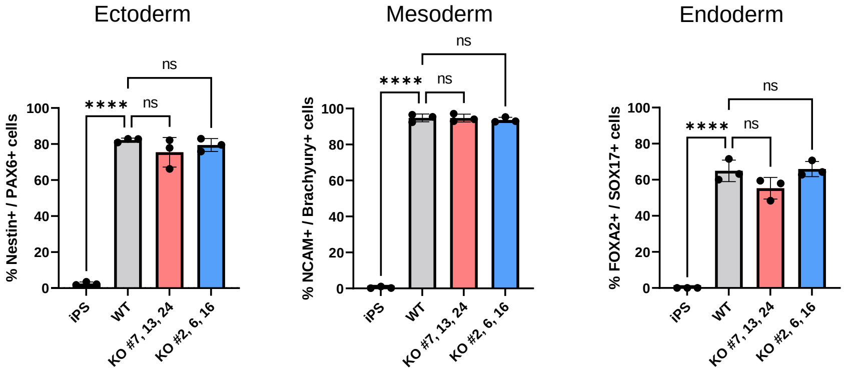
Ectoderm
Mesoderm
Endoderm
% Nestin+ / PAX6+ cells
% NCAM+ / Brachyury+ cells
% FOXA2+ / SOX17+ cells
0
20
40
60
80
100
****
ns
iPS
WT
KO #7, 13, 24
KO #2, 6, 16

Figure S4. Capacity of differentiation into three germ layers.

A) Immunofluorescence micrographs of differentiation into three germ layers. Ectodermal differentiated cells were stained using Nestin and PAX6 antibodies (top). Mesodermal differentiated cells were stained using NCAM and Brachyury antibodies (middle). Endodermal differentiated cells were stained using FOXA2 and SOX17 antibodies (bottom). All cells were stained by DAPI for nuclear staining. Scale bar, 100 μm.

B) Quantification of three germ layer marker-positive cells by flow cytometry. ns, not significant. Asterisks indicate statistical significance: **** $p < 0.0001$.

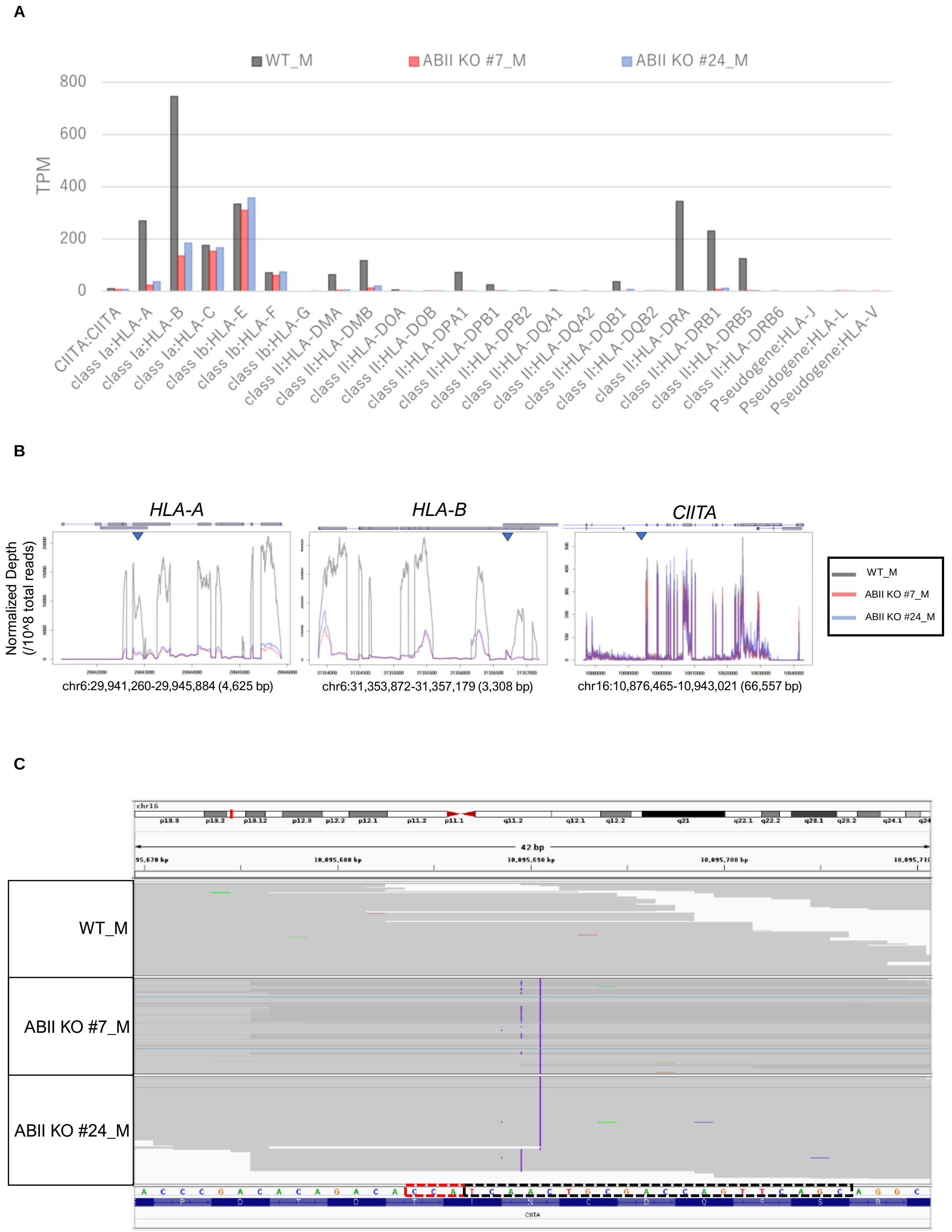

Figure S5. The mRNA expression of HLA-related genes in differentiated CD14+ monocytes derived from genome-edited iPSCs.

A) Transcripts per million (TPM) values showing the normalized gene expression of the HLA-related genes in CD14+ monocytes derived from genome-edited iPSC clones. CD14+ monocytes derived from WT and genome-edited #7 and #24 iPSCs are colored black, red and blue, respectively.

B) Diagram showing the normalized depth of the HLA-A, HLA-B and CIITA loci in differentiated monocytes derived from control and genome-edited iPSC clones (#7 and #24).

C) Integrated genome viewer (IGV) image showing the insertion of the base (purple vertical lines) in the sequence reads in ABII KO #7_M and ABII KO #24_M, which was not found in WT_M.

A

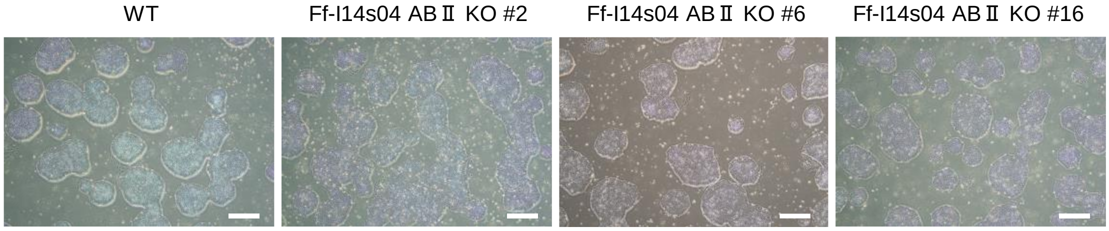

B

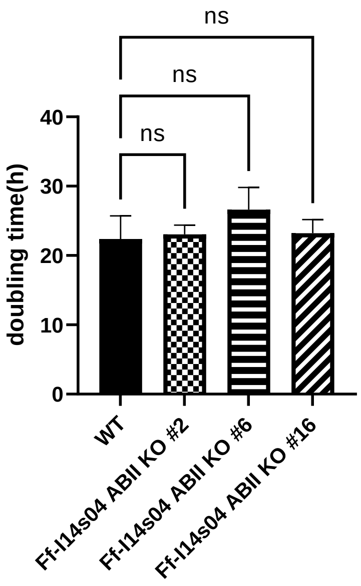

Figure S6. Differences in cell characteristics before and after genome editing.

A) Morphology of wild-type iPSC and HLA-A/B/CIITA genome-edited iPSC clones. Scale bar, 200 μm.

B) Barplot showing the doubling time of wild-type iPSC and genome-edited iPSC clones #2, #6 and #16. Three biological replicates were performed. ns, not significant.

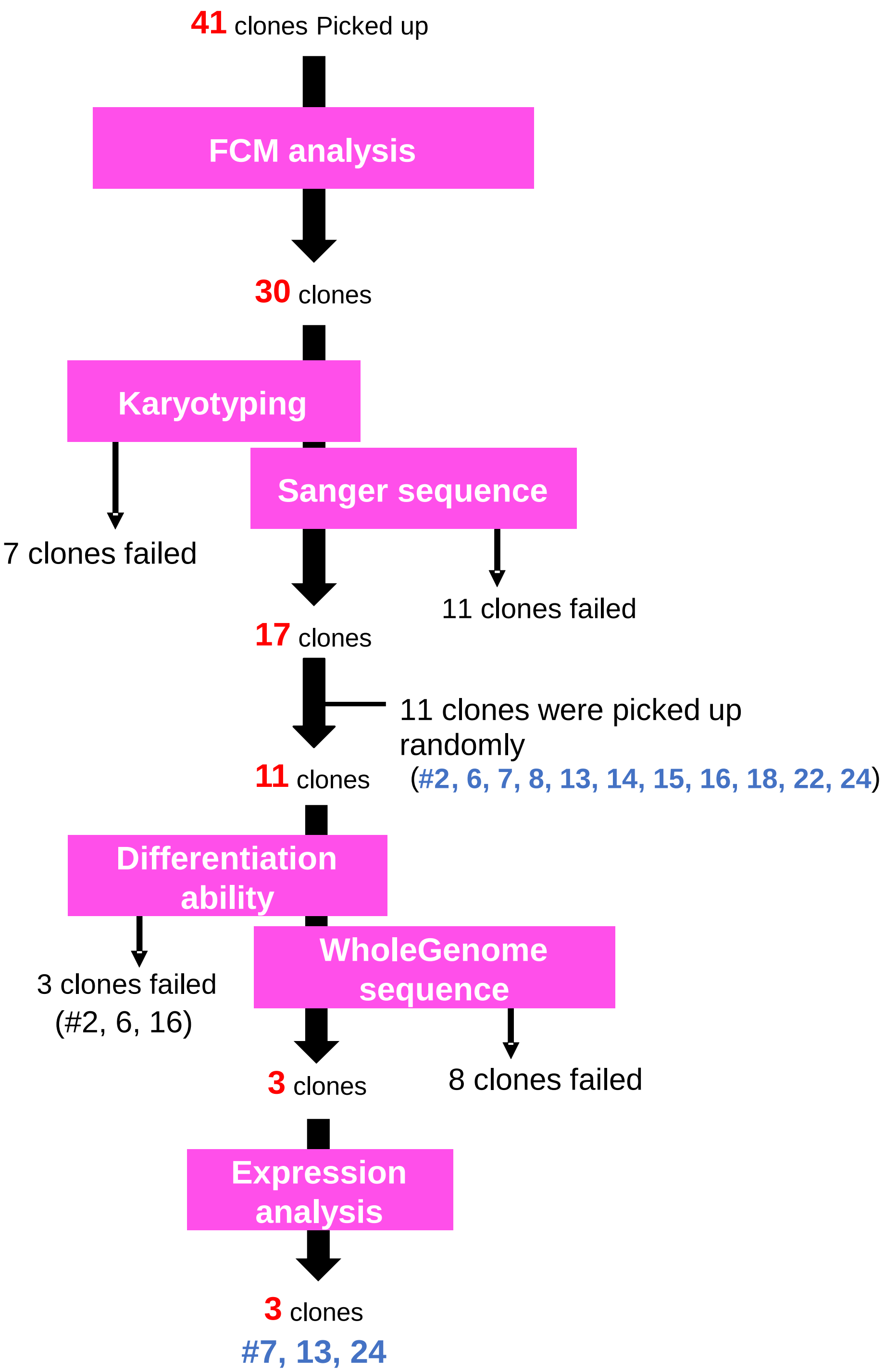

Figure S7. Summary of genome-edited iPSC clones selection.
We selected 30 clones with HLA-A$^{(-)}$/HLA-C$^{(+)}$ by FCM. We then examined the karyotype to assess genomic integrity and conducted Sanger sequencing to confirm the In/del pattern in the target regions. Thirteen clones failed this screening. Eleven clones were randomly selected for whole genome sequencing and a differentiation test. Three clones passed these tests. Finally, we assessed the gene expression level and obtained three genome-edited iPSC clones.

Table S1. HLA-A/B editable single guideRNA design.

Target gene : HLA-A and HLA-B

Target exon : exon2 or 3

PAM : NGG

Design tool : CRISPOR 4.99

Frequency ranking in Japanese population	Code of HLA-homozygous donors	HLA haplotype	Target exon	Sequences(5'-3')
1	QHJI	A*24:02<br>B*52:01	Exon 2	AGAGAACCTGCGGATCGCGC
2	RWMH	A*33:03<br>B*44:03	Exon 3	CAGGACCGCCTACGACGGCA
3	DRXT	A*24:02<br>B*07:02	Exon2	CCGTGTCCCGGCCCGGCCGC<br>GGGCTCCCCGCGGCCGGGCC<br>GCGGGCGCCGTGGATAGAGC<br>GGCCCCTCCTGCTCTATCCA
4	RJWI	A*24:02<br>B*54:01	Exon2	CGATGAAGCGGGGCTCCCCG

Table S2. Mutation patterns of target site-cloning and sequencing.

HLA-A	HLA-B	CIITA
del(deletion)-GA-re(reverse)	del-GAGAGAACC-for(forward)	del 100bp<-for
WT	ins-TCCTGCGACTTACCGC-for	del-CCTCCCAGAACCCGACACAGACACCATCAACTGCGACCAGT-for
del-CGAGA-re	WT	WT
del-CGAGA-re	del-GAACCTGC-rev	ins-A-for
del-GAGAGAACC-re	del-GA-for	del-CCATCAAC-rev
unclear	del-GA-rev	unclear
del-GAGAGAACC-re	WT	ins-A-rev
del-ACCTGCGGATCGCGCTCCGCTA-re	del-GAACCTGC-for	ins-A-for
ins(insertion)-A-re	WT	del-TGCGAC-rev
del-ACCTGCGGATCGCGCTCCGCTA-re	del-GAGAGAACC-rev	del-TCAACTGCGACCA-for
WT	del-AGAACCTG-rev	WT
		ins-AA-rev

Table S3. Amino acid predictions of the genome-edited iPSC clones.

Ff-I14s04	HLA-A		HLA-B		CIITA	
ABII KO	Allele1	Allele2	Allele1	Allele2	Allele1	Allele2
#2	c.296_304del<br>p. Arg99_Asn101del		c.300_301delGA<br>p. Asn101Profs*37 Exon3		c.220_221insA<br>p. Asn74Lysfs*11 Exon3	c.220_221insC<br>p. Asn74Thrfs*11 Exon3
#6	c.300_311del<br>p. Glu100Asp / p. Asn101_Ile104del		c.294_299del<br>p. Try98* Exon2		c.220_221insA<br>p. Asn74Lysfs*11 Exon3	
#7	c.299_300insA<br>p. Asn101Glufs*120 Exon4	c.299delA<br>p. Glu100Glyfs*22 Exone2	c.299_300insT<br>p. Glu100Aspfs*39 Exon3	c.300_306del<br>p. Glu100Aspfs*49 Exon3	c.220_221insA<br>p. Asn74Lysfs*11 Exon3	
#8	c.299_300insC<br>p. Glu100Aspfs*121 Exon4		c.300_301delGA<br>p. Asn101Profs*37 Exon3		c.220_221insA<br>p. Asn74Lysfs*11 Exon3	
#13	c.299_300insA<br>p. Asn101Glufs*120 Exon4		c.299_300insA<br>p. Asn101Glufs*38 Exon3		c.220_221insA<br>p. Asn74Lysfs*11 Exon3	
#14	c.296_304del<br>p. Arg99_Asn101del		c.300_301delGA<br>p. Asn101Profs*37 Exon3	c.289_314del<br>p. Thr97Alafs*33 Exon3	c.220_221insA<br>p. Asn74Lysfs*11 Exon3	
#15	c.283_299del<br>p. Ser95Glufs*120 Exon4		c.299_300insG<br>p. Asn101Glufs*38 Exon3		c.220_221insA<br>p. Asn74Lysfs*11 Exon3	c.220_221insT<br>p. Asn74Ilefs*11 Exon3
#16	c.299_300insA<br>p. Asn101Glufs*120 Exon4		c.299_300insC<br>p. Glu100Aspfs*39 Exon3		c.220_221insA<br>p. Asn74Lysfs*11 Exon3	c.221_228del<br>p. Asn74Thrfs*8 Exon3
#18	c.284_299del<br>p. Ser95* Exon2		c.299_300insA<br>p. Asn101Glufs*38 Exon3		c.220_221insC<br>p. Asn74Thrfs*11 Exon3	c.212_231del<br>p. Asp71Valfs*7 Exon3
#22	c.300_301delGA<br>p. Asn101Profs*119 Exon4		c.300_302delGAA<br>p. Glu100* / p. Asn101Asp		c.220_221insA<br>p. Asn74Lysfs*11 Exon3	c.220_221insAA<br>p. Asn74Lysfs*42 Exon4
#24	c.300delG<br>p. Asn101Thrfs*21 Exon2	c.300_310del<br>p. Glu100Aspfs*117 Exone4	c.299_300insA<br>p. Asn101Glufs*38 Exon3	c.300_307del<br>p. Asn101Aspfs*35 Exon3	c.220_221insA<br>p. Asn74Lysfs*11 Exon3	

Table S4. Denovo CNV mutation in genome edited cells.

ABII_ KO_2	ABII_ KO_6	ABII_ KO_7	ABII_ KO_8	ABII_ KO_13	ABII_ KO_14	ABII_ KO_15	ABII_ KO_16	ABII_ KO_18	ABII_ KO_22	ABII_ KO_24	Locus	Size(bp)	Overlap_Gene	Census (v88)	Off-target
X											chr3:128,339,436-128,341,007	1572	RPN1	RPN1	CIITA
										X	chr4:145,837,936-145,841,174	3239	-	-	
X	X						X				chr6:29,911,740-31,327,897	1416158	HLA-A,HCG9,ZNRD1-AS1,HLA-J,HCG8,ZNRD1,PPP1R11,RNF39, TRIM31,TRIM31-AS1,TRIM40,TRIM10,TRIM15,TRIM26,HCG17, HLA-L,MIR6891,HCG18,TRIM39,TRIM39-RPP21,RPP21,HLA-E, GNL1,PRR3,ABCF1,MIR877,PPP1R10,MRPS18B,ATAT1,C6orf136, DHX16,PPP1R18,NRM,MDC1,MDC1-AS1,TUBB,FLOT1,IER3,LINC00243, DDR1,MIR4640,GTF2H4,VARS2,SFTA2,DPCR1,MUC21,MUC22,HCG22, C6orf15,PSORS1C1,CDSN,PSORS1C2,CCHCR1,TCF19,POU5F1,PSORS1C3, HCG27,HLA-C,HLA-B	HLA-A, POU5F1	
								X			chr9:71,733,413-71,752,214	18802	TJP2	-	
								X			chr9:104,715,123-104,726,036	10914	-	-	
								X			chr9:105,545,015-105,566,729	21715	-	-	
								X			chr9:138,918,206-138,937,605	19400	NACC2	-	
								X			chr9:140,975,937-140,988,910	12974	CACNA1B	-	
										X	chr16:10,988,440-10,989,661	1222	CIITA	CIITA	
							X				chr17:44,442,296-44,629,670	187375	NSFP1,ARL17A,LRRC37A2	-	
	X										chr19:5,819,865-5,862,124	42260	NRTN,FUT6,FUT3,LOC101928844	-	
					X						chr21:25,118,166-25,121,850	3685	-	-	

Table S5. Denovo SNV mutation in genome edited cells.

ABII_ KO_2	ABII_ KO_6	ABII_ KO_7	ABII_ KO_8	ABII_ KO_13	ABII_ KO_14	ABII_ KO_15	ABII_ KO_16	ABII_ KO_18	ABII_ KO_22	ABII_ KO_24	Chr	Start	End	Ref	Alt	Func.ref Gene	Gene.ref Gene	ExonicFunc .ref Gene
x	x	x	x	x	x	x	x		x	x	chr16	10,989,545	10,989,545	-	A	exonic	CIITA	fs[a] insertion
x								x			chr16	10,989,546	10,989,546	-	C	exonic	CIITA	fs insertion
x											chr1	24,676,608	24,676,608	C	G	exonic	GRHL3	ns[b] SNV
x											chr9	19,290,830	19,290,830	C	A	exonic	DENND4C	ns SNV
x					x						chr6	29,910,752	29,910,760	GACCGAGTG	-	exonic	HLA-A	non fs deletion
	x										chr1	143,767,518	143,767,518	G	T	exonic	PPIAL4G	ns SNV
	x										chr6	28,541,552	28,541,552	G	T	exonic	ZBED9	ns SNV
	x									x	chr6	29,910,758	29,910,768	GTGGACCTGGG	-	exonic	HLA-A	fs deletion
		x									chr12	32,949,161	32,949,161	T	A	exonic	PKP2	ns SNV
		x	x	x			x				chr6	29,910,758	29,910,758	-	A	exonic	HLA-A	fs insertion
		x									chr6	31,324,502	31,324,508	CAGGCTC	-	exonic	HLA-B	fs deletion
					x						chr8	101,178,090	101,178,090	G	T	exonic	SPAG1	ns SNV
					x						chr15	72,063,533	72,063,533	G	A	exonic	THSD4	ns SNV
					x						chr18	29,264,323	29,264,325	AGA	-	exonic	B4GALT6	non fs deletion
					x						chr6	31,324,506	31,324,507	CT	-	exonic	HLA-B	fs deletion
						x					chr12	278,268	278,268	A	T	exonic	IQSEC3	ns SNV
						x					chr16	10,989,546	10,989,546	-	T	exonic	CIITA	fs insertion
						x					chr19	39,200,939	39,200,939	C	T	exonic	ACTN4	ns SNV
							x				chr12	120,762,766	120,762,766	G	A	exonic	PLA2G1B	ns SNV
							x				chr16	10,989,548	10,989,555	CTGCGACC	-	exonic	CIITA	fs deletion
								x			chr2	172,966,269	172,966,269	C	T	exonic	DLX2	ns SNV
								x			chr3	11,059,013	11,059,013	C	A	exonic	SLC6A1	ns SNV

								x			chr3	48,696,834	48,696,834	C	A	exonic	CELSR3	ns SNV
								x			chr16	10,989,535	10,989,554	CAGACACCATCA ACTGCGAC	-	exonic	CIITA	fs deletion
								x			chr21	46,945,842	46,945,842	C	G	exonic	SLC19A1	ns SNV
								x			chr6	29,910,754	29,910,761	CCGAGTGG	-	exonic	HLA-A	fs deletion
									x		chr3	40,464,893	40,464,893	G	A	splicing	ENTPD3	-
									x		chr12	7,288,877	7,288,877	G	C	exonic	CLSTN3	ns SNV
									x		chr16	10,989,545	10,989,545	-	A A	exonic	CIITA	fs insertion
									x		chr19	13,934,185	13,934,185	C	T	exonic	ZSWIM4	ns SNV
									x		chr4	88,535,868	88,535,868	A	G	exonic	DSPP	ns SNV
									x		chr6	29,910,758	29,910,759	GT	-	exonic	HLA-A	fs deletion
										x	chr4	73,003,798	73,003,798	G	C	exonic	NPFFR2	ns SNV
										x	chr12	6,675,434	6,675,434	C	T	exonic	NOP2	ns SNV
										x	chr6	31,324,500	31,324,507	CGCAGGCT	-	exonic	HLA-B	fs deletion

[a] fs means frameshift

[b] ns means non synonymous

Table S6. Primer Information.

PCR primer oligo name	PCR primer oligo sequence
1383D2-HLA-A-be-ex1-fwd	TTGGGGATTCCCCAACTCC
HLA-A-in7-rev	GTCCACTGTTCCGCCCAA
HLA-A24:02-ex2-fwd	CTCCCACTCCATGAGGTATTTCTC
HLA-A24:02-ex3-rev	CCCTCCAGGTAGGCTCTCT
1383D2-HLA-B-be-ex1-fwd	CAGGATACTCGTGACGCGT
1383D2-HLA-B-in7-rev	ACACACGCGAAACATCCCAA
B52-ex2-fwd	CATGAGGTATTTCTACACCGCCA
0706HLA-B-in-rev	CTGGTACCCGCGCGCTG
604B1-HLA-C-be-ex1-fwd	CAATCAGCGTCTCCGCAGT
604B1-HLA-C-ex8-rev	ATGCTAACAGGAACGCAGACA
HLA-C-ex1-fwd	TCCTGCTGCTCTCGGGAG
CIITA-Fwd	GCTTGCTGTAGAGACGGCAAT
CIITA-Rev	TTGCTCCGCATCCCCCTT
M13 forward primer	GTAAAACGACGGCCAG
M13 reverse primer	CAGGAAACAGCTATGAC

Data S1. On/off-target candidate of HLA-A/B guideRNA.

Data S2. On/off-target candidate of CIITA guideRNA.

Data S3. Statistical analysis.
