# Supplementary material for: Generation of hypoimmunogenic induced pluripotent stem cells by CRISPR-Cas9 system and detailed evaluation for clinical application
Source: Mol Ther Methods Clin Dev. 2022 May 29;26:15–25. doi: 10.1016/j.omtm.2022.05.010 (PMC9198376; doi:10.1016/j.omtm.2022.05.010)
Supplement: Document S2. Article plus supplemental information [file mmc5.pdf]

Molecular Therapy
Methods & Clinical Development
Original Article

Generation of hypoimmunogenic induced pluripotent stem cells by CRISPR-Cas9 system and detailed evaluation for clinical application

Yuko Kitano,[1] Sayaka Nishimura,[1] Tomoaki M. Kato,[1] Anna Ueda,[1] Kaho Takigawa,[1] Masafumi Umekage,[1] Masaki Nomura,[1] Ayane Kawakami,[1] Haruna Ogawa,[1] Huaigeng Xu,[2,3] Akitsu Hotta,[2] Naoko Takasu,[1] and Masayoshi Tsukahara[1]

[1]CiRA Foundation, 53 Shogoin Kawahara-cho, Sakyo-ku, Kyoto 606-8397, Japan; [2]Center for iPS Cell Research and Application, Kyoto University, 53 Shogoin Kawahara-cho, Sakyo-ku, Kyoto 606-8507, Japan; [3]Eli and Edythe Broad Center of Regeneration Medicine and Stem Cell Research, University of California, San Francisco, 35 Medical Center Way, San Francisco, CA 94143, USA

In order to expand the promise of regenerative medicine using allogeneic induced pluripotent stem cells (iPSCs), precise and efficient genome editing of human leukocyte antigen (HLA) genes would be advantageous to minimize the immune rejection caused by mismatches of HLA type. However, clinical-grade genome editing of multiple HLA genes in human iPSC lines remains unexplored. Here, we optimized the protocol for good manufacturing practice (GMP)-compatible CRISPR-Cas9 genome editing to deplete the three gene locus (HLA-A, HLA-B, and CIITA genes) simultaneously in HLA homozygous iPSCs. The use of HLA homozygous iPSCs has one main advantage over heterozygous iPSCs for inducing biallelic knockout by a single gRNA. RNA-seq and flow cytometry analyses confirmed the successful depletion of HLAs, and lineage-specific differentiation into cardiomyocytes was verified. We also confirmed that the pluripotency of genome-edited iPSCs was successfully maintained by the three germ layers of differentiation. Moreover, whole-genome sequencing, karyotyping, and optical genome mapping analyses revealed no evident genomic abnormalities detected in some clones, whereas unexpected copy number losses, chromosomal translocations, and complex genomic rearrangements were observed in other clones. Our results indicate the importance of multidimensional analyses to ensure the safety and quality of the genome-edited cells. The manufacturing and assessment pipelines presented here will be the basis for clinical-grade genome editing of iPSCs.

INTRODUCTION

Since the invention of human induced pluripotent stem cells (iPSCs) in 2007,[1,2] many physicians and researchers worldwide have considered the potential application of iPSCs in regenerative medicine to restore functionally impaired tissues or organs and have been working vigorously toward the realization of this goal. Several leading projects have already come to the stage of clinical trials for diseases such as age-related macular degeneration, Parkinson's disease and dilated cardiomyopathy,[3–5] and further clinical trials for many other diseases are under development, just as iPS cell-derived CAR-NK therapy is being adapted for tumors.[6,7]

To expand the applicability of iPSC-mediated cell therapy, the allogeneic transplantation setting is advantageous in terms of its lower manufacturing costs and readiness for patients as an off-the-shelf cell therapy product. However, immune rejection caused by mismatches of human leukocyte antigens (HLAs) between donor and recipient is a hurdle for the development of allogeneic cell therapy.[8] To overcome this issue, we have established iPSC stocks derived from healthy donors who are homozygous for HLA genes. To ensure the quality and safety of iPSC stocks, we have applied stringent selection criteria for established iPSC lines to reduce the risk of tumorigenicity due to genomic mutations, and these iPSC stocks have already been used in some of the aforementioned clinical trials.[9] It is, however, estimated that more than 140 lines of iPSCs that are homozygous for HLA-A, HLA-B, and HLA-DR will be needed to cover 90% of the Japanese population.[10] To cover multiethnic populations, the number of required lines would be even greater.[11] Thus, in addition to the establishment of HLA homozygous iPSC lines, an alternative strategy is expected to avoid the immune rejection issue.

Recent progress in genome-editing technologies has revolutionized our ability to edit the genomic sequence of iPS cells at will. In fact, several groups have demonstrated that HLA or HLA-related genes (such as the B2M gene) can be removed or inserted to modulate T cell- or NK cell-mediated immunity against iPS cells.[12–14] Accordingly, we recently developed a new strategy for the reduction of immune rejection using CRISPR-Cas9 genome editing to selectively knockout the HLA-A and HLA-B genes while retaining HLA-C

Received 22 December 2021; accepted 25 May 2022;
https://doi.org/10.1016/j.omtm.2022.05.010.

Correspondence: Masayoshi Tsukahara, Research and Development Center, CiRA Foundation, 53 Shogoin Kawahara-cho, Sakyo-ku, Kyoto 606-8397, Japan.
E-mail: masayoshi.tsukahara@cira-foundation.or.jp

This is an open access article under the CC BY license (http://creativecommons.org/licenses/by/4.0/).

and other non-classical HLAs.[15] If necessary, the CIITA (class II, major histocompatibility complex, transactivator) gene can also be knocked out to suppress the expression of HLA class II genes. Such HLA genome-edited iPSCs could escape from both killer T cells and NK cells by deleting HLA-A, HLA-B, and all class II HLAs. The retained HLA-C with a haplotype mismatch can therefore be an antigen for T cells.[16] Hence, we propose to match the HLA-C haplotype with donors. Due to the lower diversity of the HLA-C haplotypes compared with that of HLA-A or HLA-B genes, we estimated that seven lines of HLA-edited iPSCs would thus cover >95% of the Japanese population, while 12 lines would cover >90% of multiethnic populations.[15]

At the same time, however, it is reported that CRISPR genome editing may induce unintended genomic mutations at off-target sites,[17,18] or unexpected large deletions at on-target sites.[19] The CRISPR system uses a guide RNA (gRNA) to recognize the target site; however, some mismatches can be tolerated. Hence, when a similar target sequence with one or more mismatch bases exists elsewhere in the human genome, such off-target sites may be cleaved and a mutation might be induced. There is also a risk of chromosomal translocation when double-strand breaks are introduced into different chromosomes.[20,21] Multiplexed gene disruption is necessary for the generation of hypoimmunogenic iPSCs. In addition, the DNA sequences of the HLA genes are highly similar to each other, hence the risk of off-target mutagenesis or chromosomal rearrangement could be higher than when targeting another gene locus. The risk associated with the clinical translation of multiplexed HLA gene editing remains to be explored.

To translate this HLA editing strategy for clinical applications, we established the manufacturing procedures for CRISPR-Cas9 genome editing in iPSCs under good manufacturing practice (GMP)-compliant conditions. Since HLA genes are highly variable among individuals and between the two alleles, it is technically challenging to design a proper gRNA that can target both alleles of the HLA gene, while avoiding the risk of off-target binding. To this end, we took advantage of our HLA homozygous iPSC lines originating from clinical-grade cell lines that we previously established, as the genomic sequences of both HLA alleles are almost identical in HLA homozygous donors. Hence, a single gRNA is sufficient for targeting both alleles, and we could avoid using multiple gRNAs for targeting a single HLA gene. For clinical-grade CRISPR-Cas9 genome editing, we used GMP-compatible 4D-Nucleofector to electroporate the complex of current GMP (cGMP)-grade Cas9 protein and chemically synthesized gRNAs into HLA homozygous iPSCs. After confirming the bulk knockout efficiency of HLA proteins by flow cytometry, we isolated subclones and performed stringent genomic integrity tests and cellular functional assays, including lineage-specific differentiation. We found that genome editing at the HLA loci could cause unexpected copy number loss, chromosomal translocation, and complex genomic rearrangement in multiple clones assessed by whole-genome sequencing (WGS), karyotyping, and optical genome mapping analyses. Our results highlight the challenges in targeting the HLA gene locus with the CRISPR-Cas9 system for future clinical applications. Therefore, we would like to propose the importance of stringent selection tests based on multidimensional assays, including rigorous genomic integrity tests, to translate the promise of hypoimmunogenic iPSCs for upcoming clinical applications.

RESULTS

Generation of hypoimmunogenic iPSCs by CRISPR-Cas9 genome editing

To establish standard operation protocols for the generation of clinical-grade hypoimmunogenic iPSCs, we started the selection of parental iPSC lines. We selected an iPSC line named Ff-I14s04, which was previously generated from peripheral blood mononuclear cells provided by a donor (ID: QHJI) carrying homozygous HLA alleles (A*24:02, B*52:01, DR*15:02), which are most common in the Japanese population.[9] In terms of the gRNA design, we used our internal genome sequence data for this cell line in order to verify the on-target specificity of a given gRNA. Due to the high homology between the HLA-A and HLA-B genes, we found that simultaneous genome editing of HLA-A and HLA-B genes using a single gRNA could be feasible (Table S1). Therefore, we designed one gRNA (HLA-A24-ex2g1) that targets both the HLA-A and HLA-B genes on chromosome 6, and another gRNA (CIITA-ex3g5) that targets the CIITA gene on chromosome 16 (Figure 1A). After optimization of the electroporation conditions, we electroporated the Cas9 protein and two gRNAs (HLA-A24-ex2g1 and CIITA-ex3g5) into the Ff-I14s04 clone by using 4D-Nucleofector. The knockout efficiency of the HLA-A and HLA-B protein in the bulk cell population was confirmed by HLA-A or B antibody staining and flow cytometry (FCM), after interferon (IFN)-γ stimulation for 2 days. The results showed that 77.9% of the cells were negative for the cell surface expression of HLA-A protein and 72.1% were negative for HLA-B protein, suggesting that biallelic knockout was achieved (Figure 1B).

Furthermore, to examine the insertion or deletion (indel) efficiency of HLA-A, HLA-B, and CIITA target sites in more detail, target-site cloning and sequencing were performed. Approximately 80% of target sites had indel mutations (Figure S1A and Table S2). The fact that the efficiency of biallelic knockout obtained from FCM, and indel induction obtained from the genomic DNA sequences of each of the three genes were almost comparable suggested that success or failure of the genome editing against the three loci by two gRNAs in each cell were synchronized rather than independent. Next, we performed limiting dilution of the bulk edited cells and seeded cells into multiple 96-well plates at a density of 0.25 cells/well. After isolation and expansion of the single-cell-derived clones up to 6-well plates, we assessed the HLA-A protein expression by flow cytometry of each clone after IFN-γ treatment. According to a flow cytometry experiment using an HLA-A antibody after cloning, we selected 30 clones of HLA-A knockout (KO) iPSCs from 41 analyzed clones (73.1%). We further subjected these clones to Sanger sequencing, karyotyping, WGS, RNA and protein expression analyses, and assessment of differentiation potential (Figure 1C). When we first tried to amplify the HLA-A, HLA-B, and CIITA regions of 30 subclones by

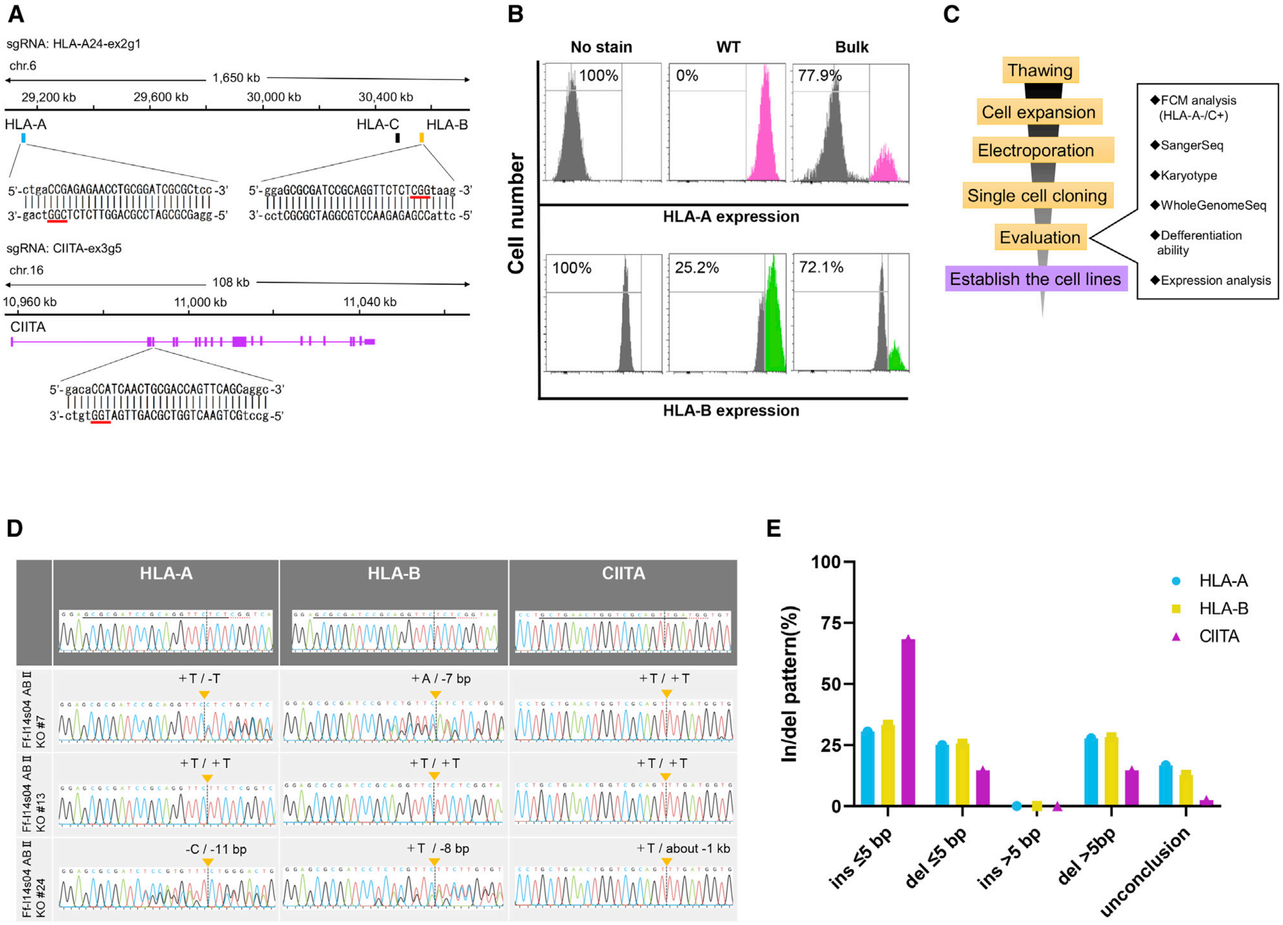

Figure 1. Generation and evaluation of simultaneous knockout of the HLA-A, HLA-B, and CIITA genes in iPSCs by CRISPR-Cas9

(A) The design of a gRNA (HLA-A24-ex2g1) to simultaneously target the HLA-A and HLA-B genes, and another gRNA (CIITA-ex3g5) to target the CIITA gene. The genomic coordinate is based on hg19. (B) Histograms of the immunoreactivity intensity of the anti-HLA-A and anti-HLA-B antibodies for bulk genome-edited iPSCs. (C) The workflow of the production of genome-edited iPSC clones and their evaluation. (D) Sanger sequencing of the genome-edited iPSCs after single-cell cloning. Insertions or deletions at the target sites in the genome-edited iPSC clones were confirmed by electropherograms. (E) Size distribution of insertions or deletions at each target gene locus.

PCR, we either could not obtain PCR products, or we obtained PCR products that differed greatly from the predicted product size in 11 clones (Figure S1B). We found chimeric sequences involving HLA-A and HLA-B in four clones (Figure S1C). These results suggested the existence of global genomic rearrangement involving the targeted loci.

Sanger sequencing of the HLA-A, HLA-B, and CIITA regions revealed that 19 iPSC clones among the above 30 clones had indel mutations, not only at the HLA-A locus but also at the HLA-B and CIITA loci, and six clones had an in-frame mutation in at least one of the HLA-A or HLA-B loci (Figures 1D and S1D). Among these mutations, almost half of the iPSC clones had indels of 5 base pairs (bp) or longer in the HLA loci, whereas 80% of the clones had indels of less than 5 bp in the CIITA locus (Figure 1E). Therefore, the sizes of the indel mutations may be affected by the sequences of the gRNAs or target regions.[22,23] The predicted amino acid sequences of the genome-edited iPSC clones—with the exception of clones possessing in-frame mutations—were shortened by the induction of premature stop codons within exons 2 to 4 (Table S3).

Comprehensive analysis of genetic mutations caused by the CRISPR-Cas9 system

To reveal the mutations that occurred during the genome editing and clonal isolation process, we conducted short read-based WGS, karyotyping, and optical genome mapping using genome-edited iPSC clones. First, we compared the aligned sequences obtained from genome-edited iPSC clones with their parental (wild-type) iPSCs after WGS. The copy number analysis indicated that three (Ff-I14s04 ABII KO #2, #6 and #16) of 11 randomly selected iPSC clones that were subjected to WGS harbored hemizygous large deletions of approximately 1.4 Mbp between the HLA-A and HLA-B loci (Figure 2A). In addition to these three iPSC clones, we identified chimeric

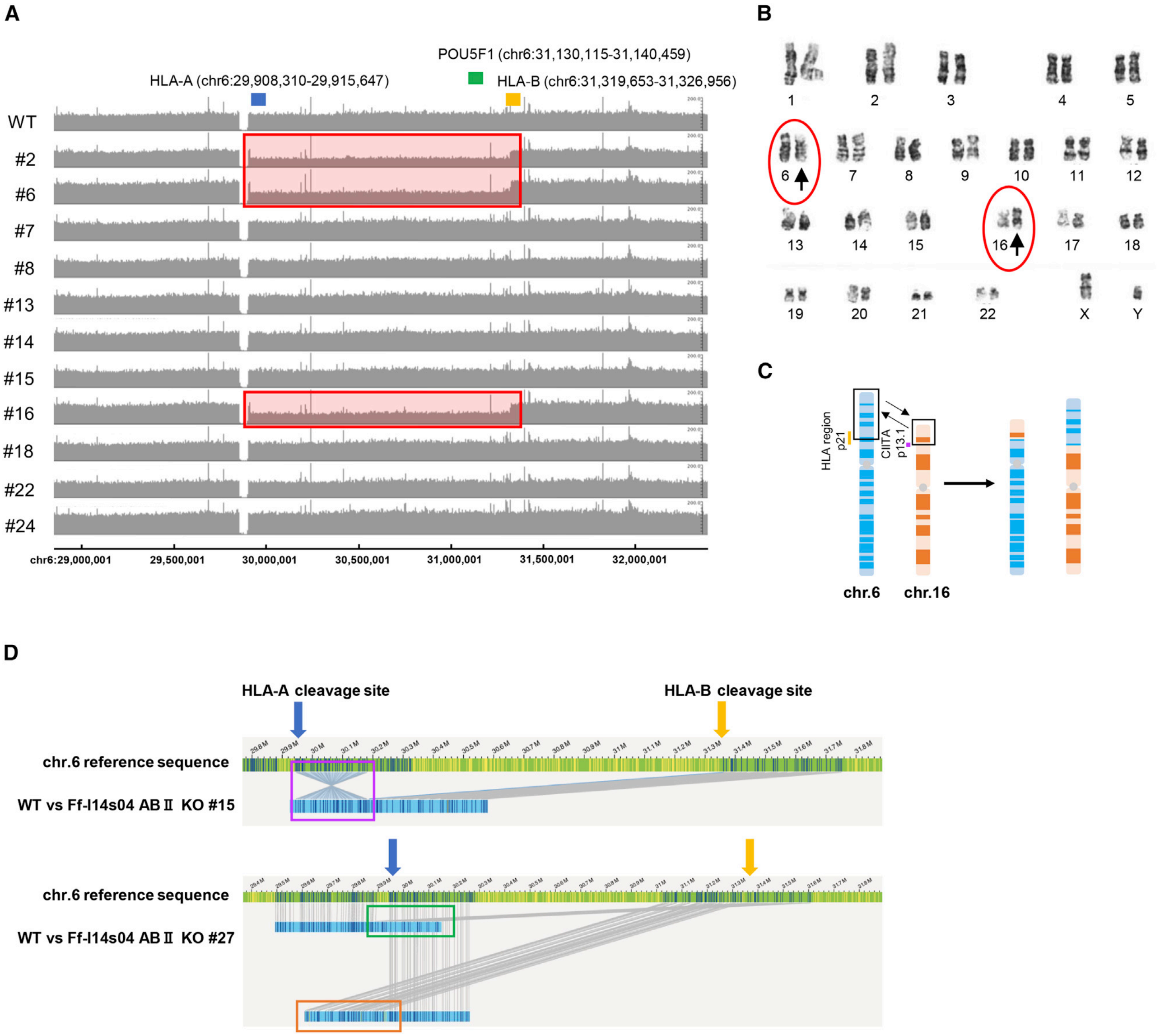

Figure 2. Genomic integrity of genome-edited iPSC clones

(A) A copy number analysis from whole-genome sequencing at the HLA class I gene cluster on the chromosome (chr) 6. The locations of HLA-A (blue), HLA-B (orange), and POU5F1 (green) are indicated above. Out of 11 clones analyzed, three clones showed a copy number loss between HLA-A and HLA-B genes (chr6: 29,911,740–31,327,897), indicated by a red box. (B) A karyotype analysis by G-banding of the genome-edited iPSCs. (C) The diagram shows a model of translocation between chr 6 and chr 16, which was found in seven iPSC clones among the 30 clones we tested. (D) Complicated genomic rearrangements were observed in optical genome mapping. Blue bars show the mutated chromosome regions. The genomic coordinate is hg38.

sequence reads in three iPSC clones (Ff-I14s04 ABII KO #8, #18 and #22) at the HLA-A and HLA-B loci, indicating that unexpected genomic rearrangement occurred in the process of repair after genomic digestion by the CRISPR-Cas9 system. Among the other mutations found in the copy number analysis, a mutation located on chromosome (chr)3 in Ff-I14s04 ABII KO #2 might have been included in a possible CIITA gRNA off-target region, which was defined by an interval of 1 kbp around the sequences, allowing for 5 bp of mismatches and/or gaps and including the RPN1 gene, which was listed among known cancer-related genes (referred to in COSMIC census version 88, https://cancer.sanger.ac.uk/cosmic/census, Table S4, Data S1 and S2). We further investigated small mutations, including single nucleotide variants (SNVs) and small indels, by comparing genome-edited iPSCs with wild-type iPSCs. The selected SNVs and indels potentially affected amino acid sequences that were not located in either genes related to cancer (other than the HLA-A and CIITA genes) or predicted off-target regions (Table S5).

Next, the karyotyping results demonstrated that seven genome-edited iPSC clones among the 30 clones showed chromosomal translocations between chr6 (HLA locus) and chr16 (CIITA locus) (Figures 2B and 2C). For a more detailed analysis, we subjected nine genome-edited iPSC clones, including wild-type iPSCs (as controls) to optical genome mapping. The results of this analysis indicated that Ff-I14s04 ABII KO #8 and #12 presented chromosomal translocations between HLA-A or HLA-B and CIITA sites (Figure S2), which were consistent with the results of the karyotyping test, whereas Ff-I14s04 ABII KO #15 and #27 harbored complex mutations that included partial translocation and inversion near the HLA-A and HLA-B cleavage sites (Figure 2D). All of these comprehensive genetic analyses revealed the frequent occurrence of unexpected genomic rearrangement caused by simultaneous triple gene editing of HLA-A, HLA-B, and CIITA.

Evaluation of the differentiation potential of genome-edited iPSC clones

Next, to confirm whether genome-edited iPSC clones retained similar differentiation potential to the original iPSC clones, we examined the efficacy of cardiomyocyte differentiation. After the induction of cardiomyocytes though embryo body (EB) formation and a three-stage stepwise differentiation protocol for 15 days, the percentage of troponin T (TnT)-positive cells was measured by FCM (Figures 3A and S3A). The original iPSC clone and eight of the 11 whole-genome-analyzed HLA-edited iPSC clones that were subjected to differentiation assays showed a >50% TnT-positive cell percentage after the induction of differentiation, whereas the other three clone Ff-I14s04 ABII KO #2, #6, and #16 showed a low TnT-positive cell ratio (Figure 3B). As shown in Figure 2A, these three iPSC clones have hemizygous large deletions between the HLA-A and HLA-B loci. More than 30 genes are located between the HLA-A and HLA-B loci, including the POU5F1 (alternatively OCT3/4) gene, which is one of the most important genes for the maintenance of pluripotency. The expression analysis of these genome-edited iPSCs using microarrays revealed that the POU5F1 expression levels in the Ff-I14s04 ABII KO #2 and #6 clones were reduced by approximately half in comparison with those in the wild-type and other genome-edited iPSC clones (Figure 3C). No significant differences were observed in the expression of other pluripotent marker genes between the clones (Figure S3B). Although the expression of many other genes was altered to a greater extent than that of POU5F1 (Figure 3D), the most plausible cause of the loss of the cardiomyocyte differentiation potential in these clones was likely the deletion of the region including POU5F1. Changes in the transcriptional profile, including the decreased expression of POU5F1, could have a large effect on the myocardial differentiation efficiency. In addition, differentiation assays of three germ layers were performed using the STEMdiff Trilineage Differentiation Kit. Immunocytochemistry and FCM were performed on the wild-type, genome-edited iPSC clones (#7, 13, 24), and those with reduced a POU5F1 expression (#2, 6, 16) to assess the differentiation status (Figure S4). There were no significant differences in the potential to differentiate into the early stage of three germ layers among wild-type and genome-edited iPSC clones or genome-edited iPSC clones with and without reduced expression of POU5F1. The down-regulation of the POU5F1 expression did not affect early differentiation, but it did significantly reduce the myocardial differentiation potential. Therefore, genome-edited iPSC lines with the deletion of the HLA-A/HLA-B intergenic region should be excluded from further analyses.

RNA expression analysis of class I and class II HLA genes in genome-edited iPSC clones

Since the genome-edited iPSC clones harbored frameshift indel mutations (Table S3), the immunoreactivity of the HLA-A antibody toward them was lost due to the destruction of the HLA-A antibody epitope, whereas that of the anti-HLA-C antibody was retained as intended (Figure 4A). With respect to HLA-B, antibody reactivity was also reduced (Figure 4A). Next, to confirm the effect HLA-A, HLA-B, and CIITA gene editing on the HLA gene expression, wild-type and genome-edited iPSCs were differentiated into CD14+ monocytes, which are known to express both HLA class I and class II genes. After the stepwise differentiation, the percentage of CD14+ monocytes was confirmed by FCM using an anti-CD14 antibody (Figure 4B). Then, we examined the RNA expression by RNA sequencing (RNA-seq) and the expression of the HLA-DR cell surface protein by FCM. The normalized gene expression, represented by transcripts per million values, was reduced for HLA-A, HLA-B, and classII HLA, but not for HLA-C, HLA-E, HLA-F, HLA-G, or CIITA in CD14+ monocytes derived from genome-edited iPSC clones (Figures S5A and S5B). The read mappings in the regions of the CIITA genes indicated that indel mutations induced by genome editing did not greatly affect the expression or maintenance of these transcripts (Figure S5B). CIITA is a transcriptional activator that up-regulates the HLA class II expression. To investigate the impact of CIITA KO, we assessed the expression of the HLA class II genes (HLA-DR, HLA-DQ, HLA-DP) and that were greatly reduced in comparison with the original non-edited CD14+ monocytes (Figure 4C). In addition, the cell surface presentation of the HLA-DR protein was lost in genome-edited iPSC-derived monocytes but not in wild-type monocytes (Figure 4D). Taken together, these results suggested that the function of the HLA-A, HLA-B, and CIITA genes was lost in genome-edited iPSC clones.

DISCUSSION

In this study, we demonstrated the manufacturing of hypoimmunogenic iPSCs and a detailed evaluation of their quality. Since we used iPSCs with homozygous HLA genes, the number of gRNAs required for gene editing targeting multiple loci could be reduced, irrespective of the genetic variability in this region (Figure 1A). This could be advantageous not only for the simplification of the production process but also for the reduction of genetic mutations caused by off-target effects of gRNAs. Among the identified small mutations, including SNVs and indels, which may affect amino acid sequences, the genome-edited iPSCs that we generated in this study did not show mutations either in cancer-related genes other than the target genes (including HLA-A, HLA-B, and CIITA) or in the neighboring predicted off-target sequences (Table S5; Data S1 and S2). Such effects

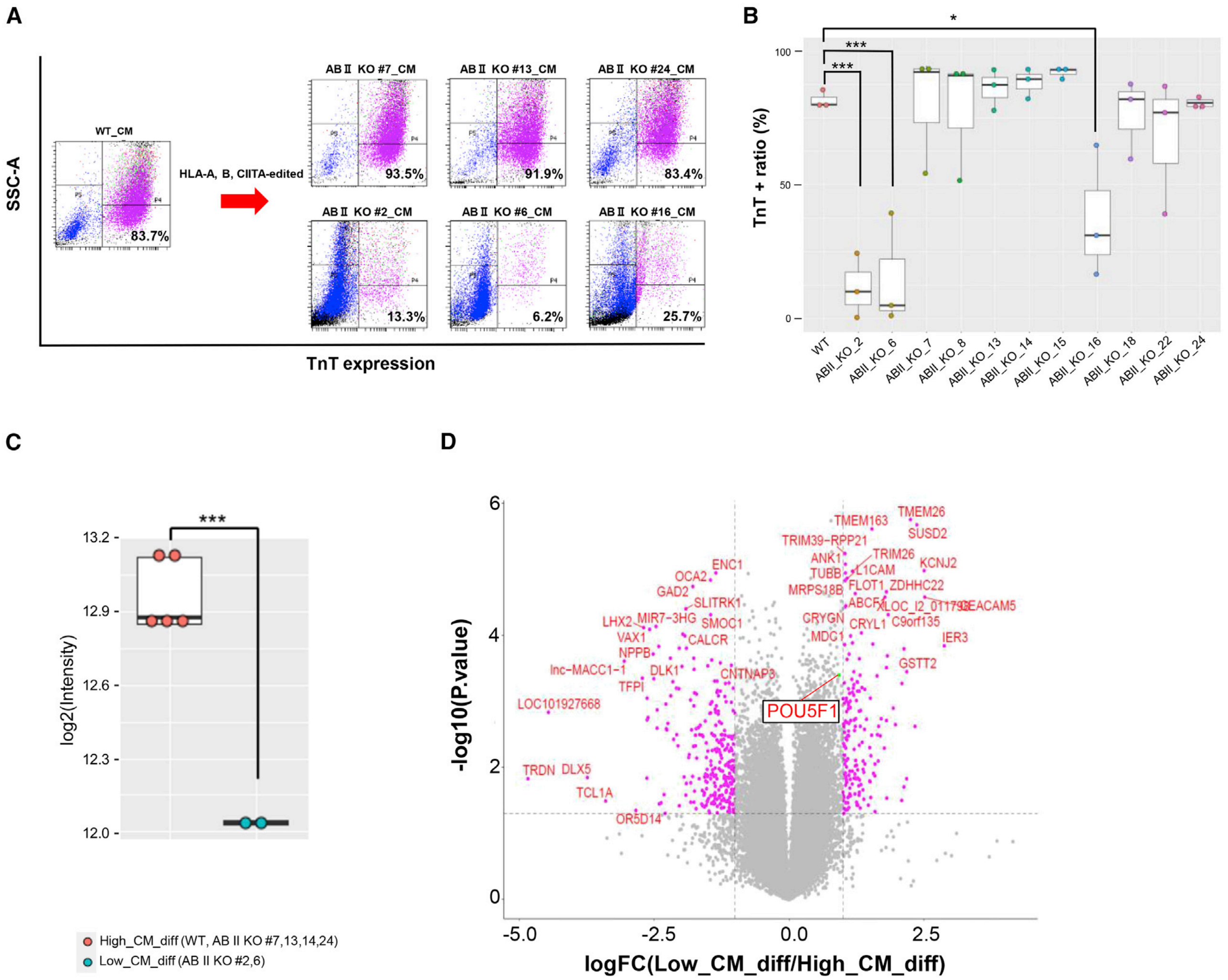

Figure 3. Evaluation of the cardiomyocyte differentiation potential of the genome-edited iPSCs

(A) Scatterplots of the FCM analysis examining the proportion of troponin T (TnT)+ cells at 15 days after the induction of cardiomyocyte differentiation. The results of the original (wild-type) and typical results of each three clones showing high or low cardiomyocyte differentiation potential. The results of other clones are shown in Figure S3A. The horizontal axis indicates the fluorescence intensities of fluorophores conjugated with the TnT antibody. The vertical axis indicates side scatter. (B) A boxplot showing the proportion of TnT+ cells in cardiomyocyte differentiation assays that were performed on three biological replicates. Asterisks indicate statistical significance: ***$p < 0.001$; *$p < 0.05$. (C) A boxplot of microarray data showing the differential expression of POU5F1 in iPSC clones showing high (orange) or low (cyan) cardiomyocyte differentiation potential. Each dot shows a clone. Asterisks indicate statistical significance: ***$p < 0.001$. (D) A volcano plot of the microarray analysis showing the differentially expressed genes between iPSC clones with high or low cardiomyocyte differentiation potential.

would be more severe in cases in which a greater number of gRNAs were used.

On the other hand, we found that more complicated mutations, such as large deletions at the intervening sequence between HLA-A and HLA-B (Figure 2A) and chromosomal translocations between the locus of HLA on chr6 and that of CIITA on chr16, often occurred in genome-edited iPSCs (Figures 2B and 2C). This might have been due to the simultaneous triple gene editing targeting HLA-A, HLA-B, and CIITA. Although step-by-step genome-editing approaches targeting individual genes at a time might reduce such mutations, it would require a longer culture period and multiple rounds of subcloning processes. In addition, repeated introduction of gRNAs and Cas9 gene or protein could be stressful for cells. Such prolonged culturing might lead to spontaneous mutagenesis that could affect the cellular characteristics. Since the detection of genome-wide low-frequency mutations in a rare cell population is technically challenging,[24] avoiding long-term culturing is desirable to reduce the risk. On the other hand, with the simultaneous targeting approach, distinctive mutations could be accurately detected by multiple genomic analyses, including optical genome mapping (Figures 2D and S2). As a result, we could avoid using iPSCs that harbor such

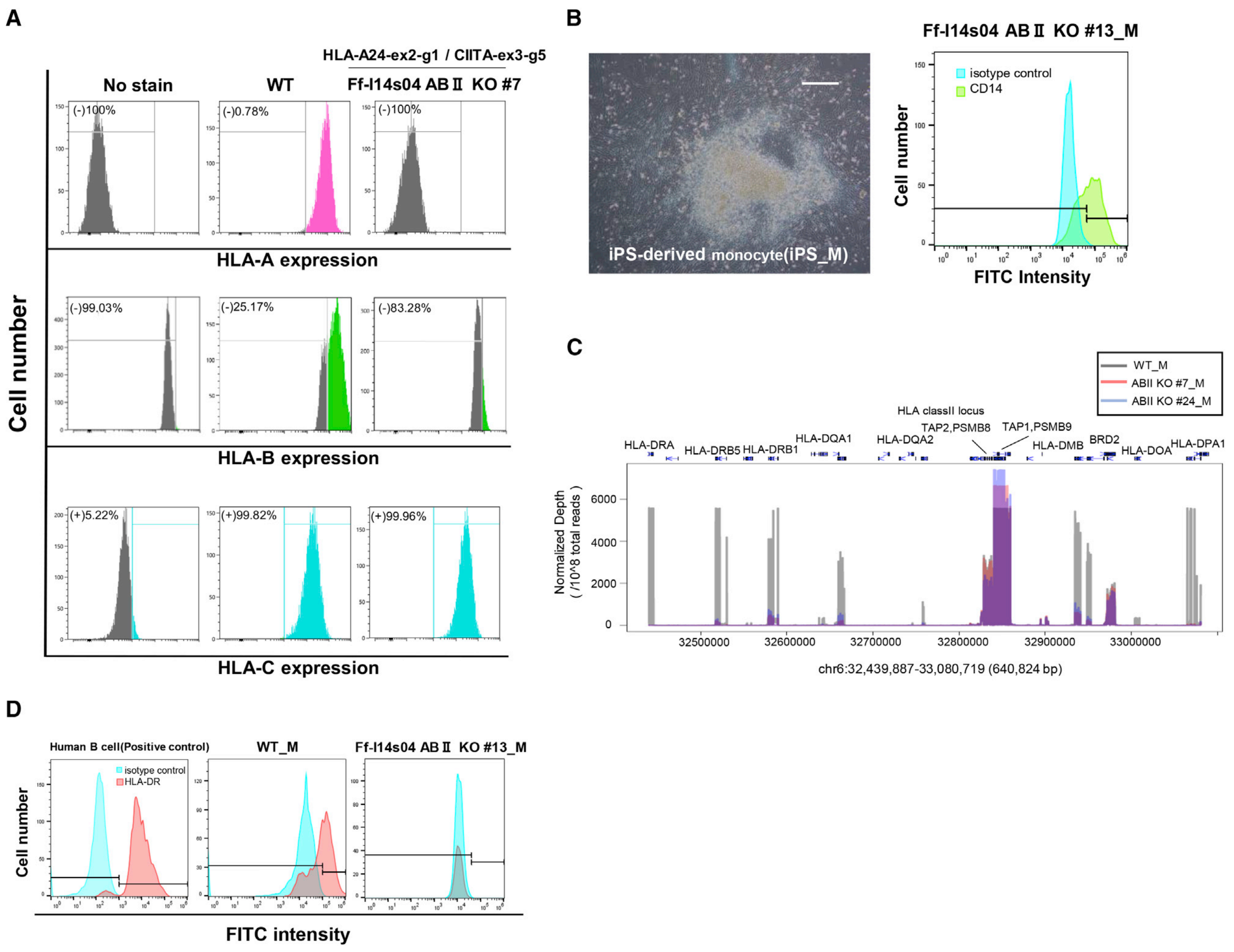

Figure 4. Validation of the expression profiles of the HLA genes in differentiated CD14+ monocytes derived from genome-edited iPSCs

(A) Flow cytometry of the original (wild-type [WT]) and clone #7, as a typical example, stained with the -HLA-A (upper panels), HLA-B (middle panels) or HLA-C (lower panels) antibodies after IFN-γ treatment. (B) Monocyte differentiation derived from the genome-edited iPSCs (clone #13, randomly selected). Scale bar, 200 μm. Flow cytometry of the cells stained with FITC-conjugated anti-CD14 antibody (green on the right panel). The same cells stained with the FITC-conjugated isotype control antibody are indicated in cyan. (C) RNA-seq data of the HLA class II locus in differentiated monocytes derived from the original (WT, gray) and genome-edited iPSC clones (#7, red and #24, blue). The normalized sequence depth (transcriptional level) from the RNA-seq data corresponding to each gene position is shown. In comparison with WT cells, HLA class II genes (HLA-DRA, DRB5, DRB1, DQA2, DMB, DOA, and DPA1) were expressed at much lower levels in genome-edited iPSC clones (#7 and #24), whereas non-HLA genes (TAP2, PSMB8, PSMB9, BRD2) were largely unchanged. (D) After monocyte differentiation of the original (WT) and genome-edited iPSCs (clone #13), flow cytometry was performed by staining with FITC-conjugated anti-class II HLA (HLA-DR, red) or FITC-conjugated isotype control antibodies after IFN-γ treatment. Human B cells were used as a positive control for HLA-DR staining.

mutations. As each genome-editing pipeline has advantages and disadvantages, the genome-editing strategy should be designed to maximize the safety of the therapeutic application.

Next, we examined whether the genome-edited iPSCs retained cardiomyocyte differentiation potential and found that the iPSCs with hemizygous large deletions at the intervening sequence between HLA-A and HLA-B (Ff-I14s04 ABII KO #2, #6, and #16) lost their differentiation potential, whereas the other iPSCs retained it (Figures 3A, 3B, and S3A). This was likely due to the hemizygous loss of alleles, including the POU5F1 gene, located between HLA-A and HLA-B (Figure S3B). POU5F1 (alternatively OCT3/4) is well known to be an important factor for the reprogramming of somatic cells.[25] Previous knockdown experiments targeting POU5F1 in human embryonic stem cells (hESCs) revealed that the reduced expression of POU5F1 led to the loss of self-renewal capacity and ectoderm or extraembryonic differentiation, but not mesoderm or endoderm differentiation.[26] In our experiment, iPSCs with the hemizygous loss of alleles, including POU5F1, could maintain their proliferation for at least 10 passages, and we could not identify any obvious characteristic morphological features of these iPSC clones, even though the POU5F1 expression levels in these iPSC clones were reduced by

half (Figure S6). Although these iPSCs lost their cardiomyocyte differentiation potential, it may be possible for the iPSCs to differentiate into other cells, such as neuronal lineages, as shown in the previous report. In fact, the reduced expression of POU5F1 did not seem to affect differentiation up to the early stage of the three germ layers (Figure S4). The situation is not exactly comparable to our iPSCs with the hemizygous loss of alleles, including POU5F1, or previous knockdown experiments in hESCs because an extremely small difference in gene expression levels can lead to different phenotypes.[27] Thus, the development of genome-editing technologies will be indispensable for the quality management of cell products for regenerative therapies. Since genome-editing technologies continue to advance rapidly, it is currently possible to precisely design experiments not only for the disruption of a gene but also for the insertion of donor sequences.[28,29] These technologies will contribute to the production of "designer cells" whose functions are modified or newly acquired from original cells other than hypoimmunogenic iPSCs.[30]

Finally, we evaluated the expression of HLA genes in CD14+ monocytes differentiated from genome-edited iPSCs at the mRNA or protein level. Frameshift indel mutations that induced a premature termination codon in CIITA did not elicit an active degradation mechanism, so-called nonsense-mediated decay (Figure S5B). Due to our incomplete understanding of the regulatory mechanisms of RNA processing and degradation, we should consider that the predicted functions after the introduction of mutations are not necessarily accurate.[31] Combinations of other assays are therefore necessary to ensure the loss of function of these genes. Regarding the CIITA gene, the expression levels of class II HLA genes, such as HLA-DRA and HLA-DRB1, were drastically reduced in CD14+ monocytes derived from genome-edited iPSCs, suggesting the loss of function of this gene (Figure 4B). In protein expression assays using FCM, we confirmed the loss of immunoreactivity of anti-HLA-A in genome-edited iPSCs and anti-HLA-DR in the antigen-presenting CD14+ monocytes derived from genome-edited iPSCs, whereas the immunoreactivity of anti-HLA-C was retained in these iPSCs (Figures 4A and 4D). Although the antibody reactivity of HLA-B was not sufficient (Figure 4A), the decrease of antibody response was certainly confirmed in genome-edited iPSCs. Furthermore, a previous study revealed that mutations that induced a premature stop codon at the α-chain of HLA molecules resulted in the loss of the HLA protein function.[32] Therefore, the HLA-B function is expected to be lost in genome-edited iPSCs with nonsense mutations in the α-chain region of HLA-B (Table S3). Here, we mainly discussed the triple gene editing approach, as we need to manage the mismatches from both class I and class II HLAs. However, caution is necessary for the CIITA knockout because CIITA is known to be involved in the transcriptional regulation for not only the class II HLAs but also other immunological genes.[33–35] Hence, when there is a concern regarding the possibility of side effects due to the loss of the CIITA, retention of the CIITA gene can be an option for some applications. Previously, Gornalusse et al.[12] reported the method that could reduce "missing-self" immune rejection response caused by NK cells. Although the overexpression of single-chain HLA-E fused with B2M could suppress NKG2A (alternatively KLRC1)+ NK cells, this approach is difficult to suppress KIR2DL1–4+ NK cell populations, as they are suppressed through HLA-C or HLA-G binding. In addition, the single-chain HLA-E fused with B2M has no antigen-presentation ability. On the other hand, our HLA-A/B knockout strategy can suppress both NKG2A+ and KIR2DL1-4+ NK cells by endogenous HLA-C or HLA-G expression. By our strategy, we started with the evaluation of 40 genome-edited iPSC clones and finally obtained only three clones that satisfied all criteria with regard to their functions and genetic mutations (Figure S7). This success rate was much lower than our initial expectation. Nonstandard products were obtained as a result of mutations that occurred due to the inappropriate repair of the digested sites introduced by simultaneous triple gene editing. As mentioned above, this may be somewhat unavoidable if we prioritize a simplified process and avoid the repetitive burden of the induction of exogenous genes. Therefore, the detailed evaluation of genome-edited cell products is important for ensuring the safety of regenerative therapies.

MATERIALS AND METHODS

Cell culture and generation of HLA-A, HLA-B, and CIITA genome-edited iPSCs

The generation of iPSCs derived from a donor who provided their informed consent and the genetic recombination experiments were approved by Kyoto University. Ff-I14s04 clones were previously generated from the peripheral blood-derived mononuclear cells of a homozygous HLA donor.[9] The culture of iPSCs under feeder-free, xeno-free conditions was performed according to the methods of a previous study.[36] The methods of gene editing targeting the HLA-A/HLA-B/CIITA genes were described in a previous study, with a few modifications.[15] Briefly, 0.3×10^6 iPSCs were transfected with an RNP (ribonucleoprotein) complex of 5 μg of GMP-grade recombinant Cas9 (Takara-bio, Shiga, Japan) and 1.25 μg of chemical synthesized gRNA (Ajinomoto Bio-Pharma) in 20 μL of Primary P3 buffer and electroporation condition program CA-137. For single-cell cloning, genome-edited iPSCs were plated on a 96-well plate by limiting dilution at 0.25 cells/well. Forty colonies that grew from single cells were picked and expanded for the following analyses.

Differentiation into the three germ layers

The differentiation of iPSCs into three germ layers was conducted using the STEMdiff Trilineage kit (STEMCELL Technologies) according to the manufacturer's protocol.

Differentiation into cardiomyocytes

Cardiomyocyte differentiation was conducted according to a previous paper with a few modifications.[37] In brief, undifferentiated iPSCs were detached and dissociated into single cells by 5-min incubation with TrypLE Select (Gibco). The cells were then suspended in StemPro34 medium supplemented with 10 μL/mL GlutaMax (Gibco), 50 μg/mL ascorbic acid, 4×10^{-4} M monothioglycerol, 150 μg/mL transferrin, 10 μM Y-27632 (WAKO, Japan), 0.5% Matrigel, and 2 ng/mL human recombinant BMP4 (R&D Systems, Minneapolis, MN) and allowed to form EBs by being placed into a

low-attachment 6-well dish and cultivated for 24 hours. On day 1, media, which included human recombinant activin A (R&D Systems), BMP4, and basic fibroblast growth factor (bFGF) (R&D Systems), were added into the wells. The final concentrations were as follows: activin A, 6 ng/mL; BMP4, 9 ng/mL; and bFGF, 5 ng/mL. On day 3, the media were changed to StemPro 34 media supplemented with 10 ng/mL vascular endothelial growth factor (VEGF) (R&D Systems), 5.4 nM SB431542, 0.06 μM Dorsomorphin, and 1 μM IWP-3, a Wnt inhibitor (Stemgent, Cambridge, MA). On day 7, the media were changed to StemPro 34 media supplemented with 10 μL/mL GlutaMax, 50 μg/mL ascorbic acid, 4×10^{-4} M monothioglycerol, 150 μg/mL transferrin, and 5 ng/mL VEGF. For the maintenance of iPSC-CMs, the culture media were refed every 2 to 3 days. On the day of cell injection, EBs were dissociated by collagenase I for 3 to 6 hours and Accumax for 10 minutes.

Differentiation into CD14+ monocytes

The differentiation of iPSCs into CD14+ monocytes was conducted using the STEMdiff monocyte kit (STEMCELL Technologies) according to the manufacturer's protocol.

Flow cytometry

The cultured iPSCs were detached from plates by adding 0.5× TripLE Select (Thermo Fisher Scientific) and 0.25 mM EDTA (Nacalai Tesque, Kyoto, Japan), after which they were washed appropriately. The iPSCs were mixed with the following diluted antibodies: anti-HLA-A24 (Clone 17A10, MBL, Japan), anti-HLA-B (Clone 0.L.6, LS Bio, USA), and anti-HLA-C (Clone DT-9, BD Biosciences, USA). iPSCs with the induction of three germ layer differentiation were fixed in 4% paraformaldehyde, permeabilized with 0.5% Triton X-100, and blocked in Blocking One (Nacalai Tesque, Kyoto, Japan). Cells were incubated with primary antibodies: anti-Nestin (Clone 10C2, CST, USA), anti-PAX6 (Clone D3A9V, CST, USA), anti-NCAM (Clone 123C3, CST, USA), anti-Brachyury (Clone D2Z3J, CST, USA), anti-FOXA2 (Clone 7E6, Abcam, UK), and anti-SOX17 (Clone D1T8M, CST, USA). They were then washed and incubated with secondary antibodies: anti-Mouse IgG Alexa Fluor Plus 555 (Thermo Fisher Scientific), anti-Mouse IgG Alexa Fluor 488 (Thermo Fisher Scientific), and anti-Rabbit IgG CF®640R (BIOTIUM). Cells after myocardial induction were fixed and permeabilized as described above, and incubated with primary antibody anti-ToroponinT (Clone 13-11; Thermo Fisher Scientific), washed, and incubated with Alexa Fluor secondary antibody described above. iPS-derived monocytes were washed appropriately, and mixed with the following diluted antibodies: anti-CD14 (Clone MEM-15, Abcam, UK) and anti-HLA-DR (Clone REA805, Miltenyi Biotec, Germany). After passing the cells through a 45-μm cell strainer (BD Japan), stained cells were applied to an SA3800 cell analyzer (SONY, Tokyo, Japan).

Immunofluorescence cell staining

iPSCs with the induction of three germ layer differentiation were prepared as described above. After incubating with the antibodies, the cells were washed three times with TBS and images were immediately captured using a Keyence microscope BZ-X810.

Target site cloning

For the analysis of mutation efficiency corresponding to each target site, genomic DNA of genome-edited bulk-iPSCs was first amplified using the HLA-A, HLA-B, and CIITA primer pairs (Table S6). Then, the PCR products were placed into the cloning site of TOPO vector by a Zero Blunt TOPO PCR cloning kit for sequencing (Thermo Fisher Scientific) and transformed into *E. coli*. More than 10 colonies of each group were checked by PCR, and Sanger sequence was performed with the M13 primers.

Sanger sequencing

Genomic DNA was used for PCR and Sanger sequencing to analyze indel patterns, which were carried out with the PCR primers shown in Table S6. Nested PCR was conducted to precisely amplify the target HLA-A, HLA-B, and HLA-C loci. The obtained amplicons were used for Sanger sequencing, which was conducted by Macrogen Japan Corp or Azenta Life Sciences.

Karyotyping analyses

(G-banding) was conducted by SRL (Tokyo, Japan).

Whole-genome sequencing

Library preparation for WGS was conducted using the KAPA HyperPrep PCR-free kit for Illumina (Roche) in accordance with the manufacturer's protocol. WGS was conducted using an Illumina NovaSeq6000 system. After trimming the sequence reads using fastp 0.20.1,[38] they were mapped to the human reference genome (hg19) by BWA MEM v0.7.15.[39] All samples were within 1.6 to 2.3 billion sequenced reads, a 75.0% to 79.2% mapping rate, and 60.3 to 82.3 average depth at the genome region. The analysis of SNVs and small indels that were introduced into the iPSCs during the genome editing and the cloning process, was conducted by comparison of genome-edited iPSCs and parental iPSCs using the Genomon pipeline v2.3.0.[40] A copy number analysis was conducted using Delly 0.7.3.[41] and VarScan2 2.4.2.[42] Images of mapped reads were obtained using IGV[43] and Genome Jack (Mitsubishi Space Software, Tokyo, Japan).

Optical genome mapping

Optical genome mapping using Saphyr (Bionano genomics) was conducted by AS ONE Bioscience (Osaka, Japan). Bionano optical genome mapping was performed according to the manufacturer's instructions. Briefly, ultra-high molecular weight (UHMW) genomic DNA was isolated from 1.5×10^6 cells using the Bionano Prep SP Blood and Cell Culture DNA Isolation Kit (Bionano Genomics, San Diego, CA, #80030). DNA quantification was performed using the Qubit dsDNA BR assay kit (Thermo Fisher Scientific) with a Qubit 4.0 Fluorometer (Thermo Fisher Scientific). A total of 750 ng of UHMW DNA was labeled using the Bionano Prep Direct Label and Stain Kit (Bionano Genomics, #80005), according to the manufacturer's protocol. DNA quantification of the labeled UHMW DNA was performed using the Qubit dsDNA HR assay kit (Thermo Fisher Scientific) with a Qubit 4.0 Fluorometer. Labeled DNA at a concentration of 4 to 15 ng/μL was then loaded onto the Saphyr Chip

G2.3 (Bionano Genomics, #20366) and run on a Bionano Saphyr instrument (Bionano Genomics), targeting 100× or 300× human genome coverage by collecting 400 GB or 1,300 GB of data per sample, respectively. *De novo* assembly and variant annotation pipeline were applied using Bionano Solve version 3.6.1 with GRCh38 as a reference. Only DNA molecules with a minimum length of 150 kbp were used for the bioinformatics analysis, along with a minimum of nine labels per molecule. SV data were visualized with Bionano Access version 1.6.1. SVs were further filtered to eliminate variants observed in the Bionano control samples.

Possible off-target sites of genome editing

These were searched using GGGenome (https://gggenome.dbcls.jp/en/) with the setting allowing for 5 bp of mismatches and/or gaps.

Amino acid sequence prediction

The HLA-A, HLA-B, and CIITA amino acid sequences of the original and genome-edited iPSC clones were translated using ApE (https://jorgensen.biology.utah.edu/wayned/ape/). The translated sequences were subjected to multiple alignments using Clustal Omega (https://www.ebi.ac.uk/Tools/msa/clustalo/).

Microarray analysis

The microarray analysis was conducted using a customized SurePrint G3 human GE microarray 8 × 60K v3.0 (Design ID:083470, Agilent) in accordance with the manufacturer's protocol.

RNA-seq

TruSeq Stranded Total RNA Prep Gold (Illumina) was used for library preparation. Sequencing of the RNA-seq library was conducted on a NovaSeq6000 platform. All samples were within 335 to 445 million sequenced reads and an 83.3% to 84.7% mapping rate, as calculated by STAR. The obtained sequences were analyzed via the ENCODE long-rna-seq pipeline v2.3.4 (https://github.com/ENCODE-DCC/long-rna-seq-pipeline). The comparison of the expression among iPSCs was conducted using R v3.6.3 (https://cran.r-project.org/).

Statistical analysis

Statistical analyses were conducted using GraphPad Prism 9 and R. In the three germ layer differentiation assays, cardiomyocyte differentiation assay, and comparison of doubling time, a one-way ANOVA with Dunnett's multiple comparison test was performed by setting the original (wild-type) as a control. In the expression analysis, a two-tailed Student's t test with Benjamin-Hochberg multiple testing correction was used (Data S3).

Study approval

The iPS cell line (Ff-I14s04) used in this study was generated under written consent with approval by CiRA, Kyoto University.

SUPPLEMENTAL INFORMATION

Supplemental information can be found online at https://doi.org/10.1016/j.omtm.2022.05.010.

ACKNOWLEDGMENTS

We are grateful to Dr. Hirofumi Harashima (AS ONE) for providing technical assistance with the genomic analysis. We thank Dr. Shin Kaneko (CiRA, Kyoto University) and Dr. Peter Gee (CiRA, Kyoto University and MaxCyte Inc.) for their helpful advice. We thank Mr. Masaya Todani (CiRA, Kyoto University) for helping in the preparation of the Graphical abstract. This work was supported by a research center network for the realization of regenerative medicine of the Japan Agency for Medical Research and Development (AMED) under Grant Number JP20bm0104001h0108.

AUTHOR CONTRIBUTIONS

Y.K., M.U., H.X, A.H., N.T., and M.T. conceived the study design. Y.K., S.N., A.U., K.T., A.K., and H.O. conducted cell culture, and molecular and immunocytochemical experiments. Y.K., T.M.K., and M.N. conducted genomic analysis. T.M.K. conducted expression analysis. Y.K. and T.M.K. wrote the manuscript.

DECLARATION OF INTERESTS

The authors declare no competing interests.

REFERENCES

1. Takahashi, K., Tanabe, K., Ohnuki, M., Narita, M., Ichisaka, T., Tomoda, K., et al. (2007). Induction of pluripotent stem cells from adult human fibroblasts by defined factors. Cell *131*, 861–872. https://doi.org/10.1016/j.cell.2007.11.019.
2. Yu, J., Vodyanik, M.A., Smuga-Otto, K., Antosiewicz-Bourget, J., Frane, J.L., Tian, S., Nie, J., Jonsdottir, G.A., Ruotti, V., Stewart, R., et al. (2007). Induced pluripotent stem cell lines derived from human somatic cells. Science *318*, 1917–1920. https://doi.org/10.1126/science.1151526.
3. Mandai, M., Watanabe, A., Kurimoto, Y., Hirami, Y., Morinaga, C., Daimon, T., Fujihara, M., Akimaru, H., Sakai, N., Shibata, Y., et al. (2017). Autologous induced stem-cell–derived retinal cells for macular degeneration. N. Engl. J. Med. *376*, 1038–1046. https://doi.org/10.1056/nejmoa1608368.
4. Takahashi, J. (2021). Clinical trial for Parkinson's disease gets a green light in the US. Cell Stem Cell *28*, 182–183. https://doi.org/10.1016/j.stem.2021.01.013.
5. Deinsberger, J., Reisinger, D., and Weber, B. (2020). Global trends in clinical trials involving pluripotent stem cells: a systematic multi-database analysis. NPJ Regenerative Med. *5*, 15. https://doi.org/10.1038/s41536-020-00100-4.
6. Boyd, N.R., Tiedemann, M., Cartledge, K., Cao, M., Evtimov, V., Shu, R., Nguyen, T., Nguyen, N., Nisbet, I., Boyd, R., and Trounson, A. (2020). Off-the-shelf ipsc derived car-nk immunotherapy for solid tumors. Cytotherapy *22*, S199. https://doi.org/10.1016/j.jcyt.2020.04.067.
7. Ueda, T., Kumagai, A., Iriguchi, S., Yasui, Y., Miyasaka, T., Nakagoshi, K., Nakane, K., Saito, K., Takahashi, M., Sasaki, A., et al. (2020). Non-clinical efficacy, safety and stable clinical cell processing of induced pluripotent stem cell-derived anti-glypican-3 chimeric antigen receptor-expressing natural killer/innate lymphoid cells. Cancer Sci. *111*, 1478–1490. https://doi.org/10.1111/cas.14374.
8. Yamanaka, S. (2020). Pluripotent stem cell-based cell therapy-promise and challenges. Cell Stem Cell *27*, 523–531. https://doi.org/10.1016/j.stem.2020.09.014.
9. Umekage, M., Sato, Y., and Takasu, N. (2019). Overview: an iPS cell stock at CiRA. Inflamm. Regen. *39*, 17. https://doi.org/10.1186/s41232-019-0106-0.
10. Okita, K., Matsumura, Y., Sato, Y., Okada, A., Morizane, A., Okamoto, S., Hong, H., Nakagawa, M., Tanabe, K., Tezuka, K.-i., et al. (2011). A more efficient method to generate integration-free human iPS cells. Nat. Methods *8*, 409–412. https://doi.org/10.1038/nmeth.1591.
11. Taylor, C.J., Peacock, S., Chaudhry, A.N., Bradley, J.A., and Bolton, E.M. (2012). Generating an iPSC bank for HLA-matched tissue transplantation based on known donor and recipient HLA types. Cell Stem Cell *11*, 147–152. https://doi.org/10.1016/j.stem.2012.07.014.

12. Gornalusse, G.G., Hirata, R.K., Funk, S.E., Riolobos, L., Lopes, V.S., Manske, G., Prunkard, D., Colunga, A.G., Hanafi, L.-A., Clegg, D.O., et al. (2017). HLA-E-expressing pluripotent stem cells escape allogeneic responses and lysis by NK cells. Nat. Biotechnol. *35*, 765–772. https://doi.org/10.1038/nbt.3860.

13. Deuse, T., Hu, X., Gravina, A., Wang, D., Tediashvili, G., De, C., Thayer, W.O., Wahl, A., Garcia, J.V., Reichenspurner, H., et al. (2019). Hypoimmunogenic derivatives of induced pluripotent stem cells evade immune rejection in fully immunocompetent allogeneic recipients. Nat. Biotechnol. *37*, 252–258. https://doi.org/10.1038/s41587-019-0016-3.

14. Han, X., Wang, M., Duan, S., Franco, P.J., Kenty, J.H.-R., Hedrick, P., Xia, Y., Allen, A., Ferreira, L.M.R., Strominger, J.L., et al. (2019). Generation of hypoimmunogenic human pluripotent stem cells. Proc. Natl. Acad. Sci. U. S. A *116*, 10441–10446. https://doi.org/10.1073/pnas.1902566116.

15. Xu, H., Wang, B., Ono, M., Kagita, A., Fujii, K., Sasakawa, N., et al. (2019). Targeted disruption of HLA genes via CRISPR-Cas9 generates iPSCs with enhanced immune compatibility. Cell Stem Cell *24*, 566–578.e7. https://doi.org/10.1016/j.stem.2019.02.005.

16. Blais, M.E., Dong, T., and Rowland-Jones, S. (2011). HLA-C as a mediator of natural killer and T-cell activation: spectator or key player? Immunology *133*, 1–7. https://doi.org/10.1111/j.1365-2567.2011.03422.x.

17. Fu, Y., Foden, J.A., Khayter, C., Maeder, M.L., Reyon, D., Joung, J.K., and Sander, J.D. (2013). High-frequency off-target mutagenesis induced by CRISPR-Cas nucleases in human cells. Nat. Biotechnol. *31*, 822–826. https://doi.org/10.1038/nbt.2623.

18. Cradick, T.J., Fine, E.J., Antico, C.J., and Bao, G. (2013). CRISPR/Cas9 systems targeting β-globin and CCR5 genes have substantial off-target activity. Nucleic Acids Res. *41*, 9584–9592. https://doi.org/10.1093/nar/gkt714.

19. Kosicki, M., Tomberg, K., and Bradley, A. (2018). Repair of double-strand breaks induced by CRISPR-Cas9 leads to large deletions and complex rearrangements. Nat. Biotechnol. *36*, 765–771. https://doi.org/10.1038/nbt.4192.

20. Choi, P.S., and Meyerson, M. (2014). Targeted genomic rearrangements using CRISPR/Cas technology. Nat. Commun. *5*, 3728. https://doi.org/10.1038/ncomms4728.

21. Ghezraoui, H., Piganeau, M., Renouf, B., Renaud, J.-B., Sallmyr, A., Ruis, B., Oh, S., Tomkinson, A.E., Hendrickson, E.A., Giovannangeli, C., et al. (2014). Chromosomal translocations in human cells are generated by canonical nonhomologous end-joining. Mol. Cell *55*, 829–842. https://doi.org/10.1016/j.molcel.2014.08.002.

22. Bae, S., Kweon, J., Kim, H.S., and Kim, J.-S. (2014). Microhomology-based choice of Cas9 nuclease target sites. Nat. Methods *11*, 705–706. https://doi.org/10.1038/nmeth.3015.

23. van Overbeek, M., Capurso, D., Carter, M.M., Thompson, M.S., Frias, E., Russ, C., et al. (2016). DNA repair profiling reveals nonrandom outcomes at Cas9-mediated breaks. Mol. Cell *63*, 633–646. https://doi.org/10.1016/j.molcel.2016.06.037.

24. Yoshihara, M., Hayashizaki, Y., and Murakawa, Y. (2017). Genomic instability of iPSCs: challenges towards their clinical applications. Stem Cell Rev. Rep. *13*, 7–16. https://doi.org/10.1007/s12015-016-9680-6.

25. Kim, K.P., Wu, Y., Yoon, J., Adachi, K., Wu, G., Velychko, S., MacCarthy, C.M., Shin, B., Röpke, A., Arauzo-Bravo, M.J., et al. (2020). Reprogramming competence of OCT factors is determined by transactivation domains. Sci. Adv. *6*, eaaz7364. https://doi.org/10.1126/sciadv.aaz7364.

26. Wang, Z., Oron, E., Nelson, B., Razis, S., and Ivanova, N. (2012). Distinct lineage specification roles for NANOG, OCT4, and SOX2 in human embryonic stem cells. Cell Stem Cell *10*, 440–454. https://doi.org/10.1016/j.stem.2012.02.016.

27. Veitia, R.A., Bottani, S., and Birchler, J.A. (2013). Gene dosage effects: nonlinearities, genetic interactions, and dosage compensation. Trends Gen. *29*, 385–393. https://doi.org/10.1016/j.tig.2013.04.004.

28. Li, P.P., and Margolis, R.L. (2021). Use of single guided Cas9 nickase to facilitate precise and efficient genome editing in human iPSCs. Sci. Rep. *11*, 9865. https://doi.org/10.1038/s41598-021-89312-2.

29. Hyodo, T., Rahman, M.L., Karnan, S., Ito, T., Toyoda, A., Ota, A., Wahiduzzaman, M., Tsuzuki, S., Okada, Y., Hosokawa, Y., and Konishi, H. (2020). Tandem paired nicking promotes precise genome editing with scarce interference by p53. Cell Rep. *30*, 1195–1207.e7. https://doi.org/10.1016/j.celrep.2019.12.064.

30. Guilak, F., Pferdehirt, L., Ross, A.K., Choi, Y.-R., Collins, K., Nims, R.J., Katz, D.B., Klimak, M., Tabbaa, S., and Pham, C.T.N. (2019). Designer stem cells: genome engineering and the next generation of cell-based therapies. Orthopaedic Research Society *37*, 1287–1293. https://doi.org/10.1002/jor.24304.

31. Tuladhar, R., Yeu, Y., Tyler Piazza, J., Tan, Z., Rene Clemenceau, J., Wu, X., Barrett, Q., Herbert, J., Mathews, D.H., Kim, J., et al. (2019). CRISPR-Cas9-based mutagenesis frequently provokes on-target mRNA misregulation. Nat. Commun. *10*, 4056. https://doi.org/10.1038/s41467-019-12028-5.

32. Elsner, H.-A., and Blasczyk, R. (2004). Immunogenetics of HLA null alleles: implications for blood stem cell transplantation. Tissue Antigens *64*, 687–695. https://doi.org/10.1111/j.1399-0039.2004.00322.x.

33. Nagarajan, U.M., Bushey, A., and Boss, J.M. (2002). Modulation of gene expression by the MHC class II transactivator. J. Immunol. *169*, 5078–5088. https://doi.org/10.4049/jimmunol.169.9.5078.

34. Devaiah, B.N., and Singer, D.S. (2013). CIITA and its dual roles in MHC gene transcription. Front. Immunol. *4*, 476. https://doi.org/10.3389/fimmu.2013.00476.

35. Bruchez, A., Sha, K., Johnson, J., Chen, L., Stefani, C., McConnell, H., Gaucherand, L., Prins, R., Matreyek, K.A., Hume, A.J., et al. (2020). MHC class II transactivator CIITA induces cell resistance to Ebola virus and SARS-like coronaviruses. Science *370*, 241–247. https://doi.org/10.1126/science.abb3753.

36. Nakagawa, M., Taniguchi, Y., Senda, S., Takizawa, N., Ichisaka, T., Asano, K., Morizane, A., Doi, D., Takahashi, J., Nishizawa, M., et al. (2014). A novel efficient feeder-free culture system for the derivation of human induced pluripotent stem cells. Sci. Rep. *4*, 3594. https://doi.org/10.1038/srep03594.

37. Funakoshi, S., Miki, K., Takaki, T., Okubo, C., Hatani, T., Chonabayashi, K., Nishikawa, M., Takei, I., Oishi, A., Narita, M., et al. (2016). Enhanced engraftment, proliferation and therapeutic potential in heart using optimized human iPSC-derived cardiomyocytes. Sci. Rep. *6*, 19111. https://doi.org/10.1038/srep19111.

38. Chen, S., Zhou, Y., Chen, Y., and Gu, J. (2018). fastp: an ultra-fast all-in-one FASTQ preprocessor. Bioinformatics *34*, i884–i890. https://doi.org/10.1093/bioinformatics/bty560.

39. Li, H., and Durbin, R. (2009). Fast and accurate short read alignment with Burrows-Wheeler transform. Bioinformatics *25*, 1754–1760. https://doi.org/10.1093/bioinformatics/btp324.

40. Shiraishi, Y., Sato, Y., Chiba, K., Okuno, Y., Nagata, Y., Yoshida, K., Shiba, N., Hayashi, Y., Kume, H., Homma, Y., et al. (2013). An empirical Bayesian framework for somatic mutation detection from cancer genome sequencing data. Nucleic Acids Res. *41*, e89. https://doi.org/10.1093/nar/gkt126.

41. Rausch, T., Zichner, T., Schlattl, A., Stütz, A.M., Benes, V., and Korbel, J.O. (2012). DELLY: structural variant discovery by integrated paired-end and split-read analysis. Bioinformatics *28*, i333–i339. https://doi.org/10.1093/bioinformatics/bts378.

42. Koboldt, D.C., Zhang, Q., Larson, D.E., Shen, D., McLellan, M.D., Lin, L., Miller, C.A., Mardis, E.R., Ding, L., and Wilson, R.K. (2012). VarScan 2: somatic mutation and copy number alteration discovery in cancer by exome sequencing. Genome Res. *22*, 568–576. https://doi.org/10.1101/gr.129684.111.

43. Thorvaldsdóttir, H., Robinson, J.T., and Mesirov, J.P. (2013). Integrative Genomics Viewer (IGV): high-performance genomics data visualization and exploration. Briefings Bioinf. *14*, 178–192. https://doi.org/10.1093/bib/bbs017.

OMTM, Volume *26*

Supplemental information

Generation of hypoimmunogenic induced pluripotent stem cells by CRISPR-Cas9 system and detailed evaluation for clinical application

Yuko Kitano, Sayaka Nishimura, Tomoaki M. Kato, Anna Ueda, Kaho Takigawa, Masafumi Umekage, Masaki Nomura, Ayane Kawakami, Haruna Ogawa, Huaigeng Xu, Akitsu Hotta, Naoko Takasu, and Masayoshi Tsukahara

A

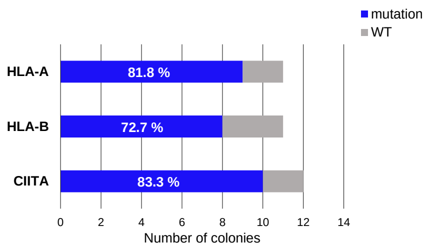
mutation
WT
HLA-A
81.8 %
HLA-B
72.7 %
CIITA
83.3 %
0 2 4 6 8 10 12 14
Number of colonies

B

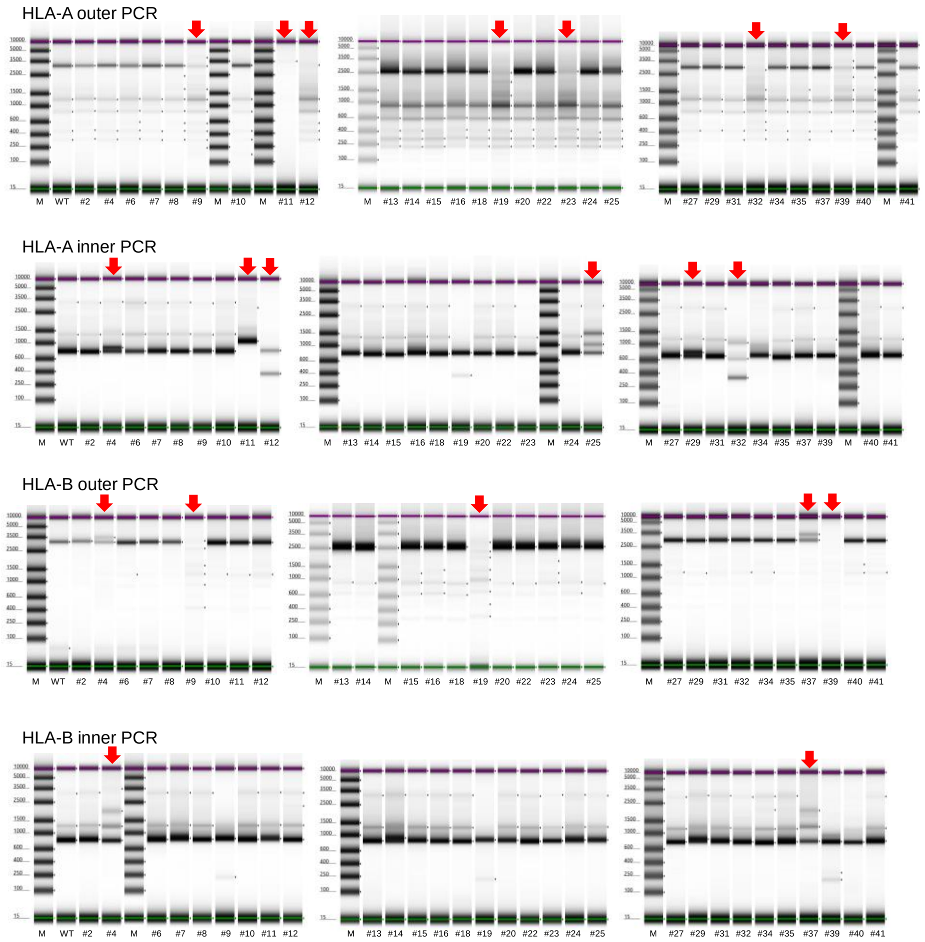
HLA-A outer PCR
M WT #2 #4 #6 #7 #8 #9 M #10 M #11 #12
M #13 #14 #15 #16 #18 #19 #20 #22 #23 #24 #25
M #27 #29 #31 #32 #34 #35 #37 #39 #40 M #41
HLA-A inner PCR
M WT #2 #4 #6 #7 #8 #9 #10 #11 #12
M #13 #14 #15 #16 #18 #19 #20 #22 #23 M #24 #25
M #27 #29 #31 #32 #34 #35 #37 #39 M #40 #41
HLA-B outer PCR
M WT #2 #4 #6 #7 #8 #9 #10 #11 #12
M #13 #14 M #15 #16 #18 #19 #20 #22 #23 #24 #25
M #27 #29 #31 #32 #34 #35 #37 #39 #40 #41
HLA-B inner PCR
M WT #2 #4 M #6 #7 #8 #9 #10 #11 #12
M #13 #14 #15 #16 #18 #19 #20 #22 #23 #24 #25
M #27 #29 #31 #32 #34 #35 #37 #39 #40 #41

C

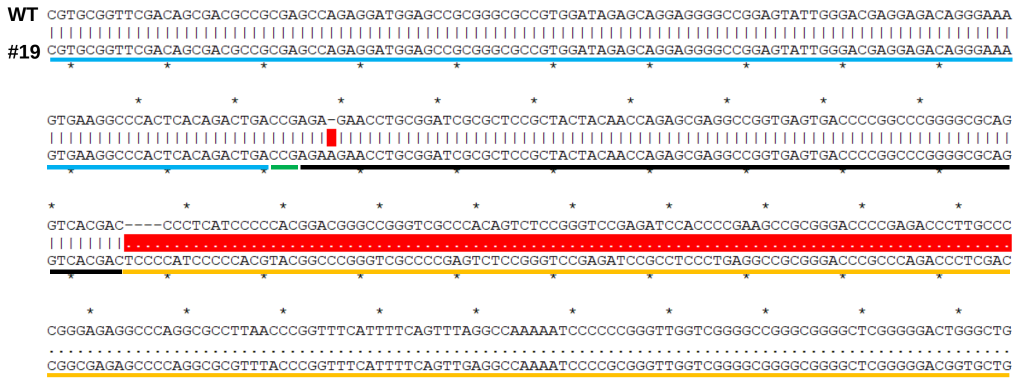
WT CGTGCGGTTCGACAGCGACGCCGCGAGCCAGAGGATGGAGCCGCGGGCGCCGTGGATAGAGCAGGAGGGGCCGGAGTATTGGGACGAGGAGACAGGGAAA
#19 CGTGCGGTTCGACAGCGACGCCGCGAGCCAGAGGATGGAGCCGCGGGCGCCGTGGATAGAGCAGGAGGGGCCGGAGTATTGGGACGAGGAGACAGGGAAA
GTGAAGGCCCACTCACAGACTGACCGAGA-GAACCTGCGGATCGCGCTCCGCTACTACAACCAGAGCGAGGCCGGTGAGTGACCCCGGCCCGGGGCGCAG
GTGAAGGCCCACTCACAGACTGACCGAGAAGAACCTGCGGATCGCGCTCCGCTACTACAACCAGAGCGAGGCCGGTGAGTGACCCCGGCCCGGGGCGCAG
GTCACGAC----CCCTCATCCCCCACGGACGGGCCGGGTCGCCCACAGTCTCCGGGTCCGAGATCCACCCCGAAGCCGCGGGACCCCGAGACCCTTGCCC
GTCACGACTCCCCATCCCCCACGTACGGCCCGGGTCGCCCCGAGTCTCCGGGTCCGAGATCCGCCTCCCTGAGGCCGCGGGACCCGCCCAGACCCTCGAC
CGGGAGAGGCCCAGGCGCCTTAACCCGGTTTCATTTTCAGTTTAGGCCAAAAATCCCCCCGGGTTGGTCGGGGCCGGGCGGGGCTCGGGGGACTGGGCTG
CGGCGAGAGCCCCAGGCGCGTTTACCCGGTTTCATTTTCAGTTGAGGCCAAAATCCCCGCGGGTTGGTCGGGGCGGGGCGGGGCTCGGGGGACGGTGCTG

D

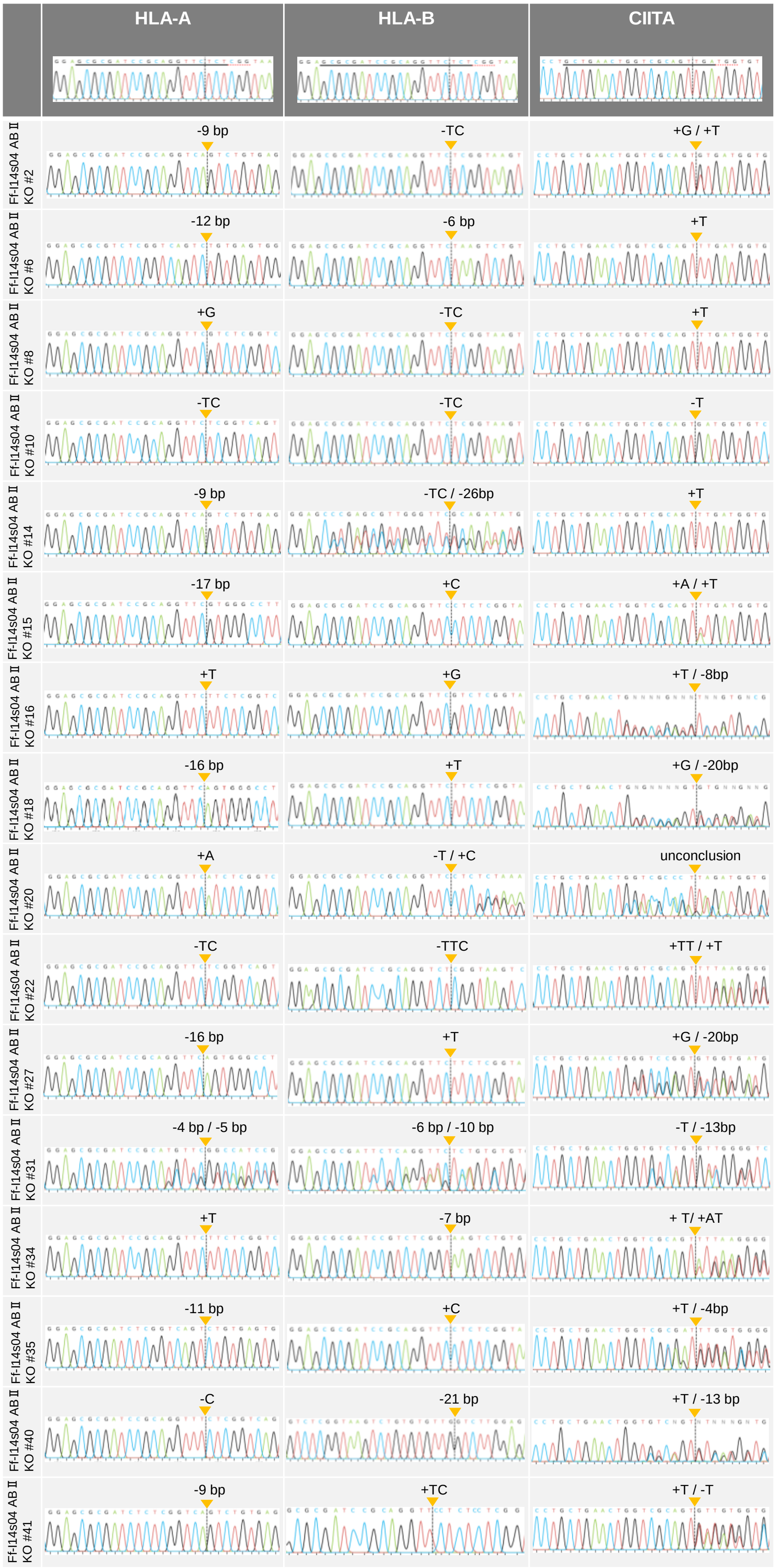
HLA-A
HLA-B
CIITA
Ff-I14s04 AB II KO #2
-9 bp
-TC
+G / +T
Ff-I14s04 AB II KO #6
-12 bp
-6 bp
+T
Ff-I14s04 AB II KO #8
+G
-TC
+T
Ff-I14s04 AB II KO #10
-TC
-TC
-T
Ff-I14s04 AB II KO #14
-9 bp
-TC / -26bp
+T
Ff-I14s04 AB II KO #15
-17 bp
+C
+A / +T
Ff-I14s04 AB II KO #16
+T
+G
+T / -8bp
Ff-I14s04 AB II KO #18
-16 bp
+T
+G / -20bp
Ff-I14s04 AB II KO #20
+A
-T / +C
unconclusion
Ff-I14s04 AB II KO #22
-TC
-TTC
+TT / +T
Ff-I14s04 AB II KO #27
-16 bp
+T
+G / -20bp
Ff-I14s04 AB II KO #31
-4 bp / -5 bp
-6 bp / -10 bp
-T / -13bp
Ff-I14s04 AB II KO #34
+T
-7 bp
+ T/ +AT
Ff-I14s04 AB II KO #35
-11 bp
+C
+T / -4bp
Ff-I14s04 AB II KO #40
-C
-21 bp
+T / -13 bp
Ff-I14s04 AB II KO #41
-9 bp
+TC
+T / -T

Figure S1. Analysis of the target site of HLA-A/HLA-B/CIITA genome-edited iPSCs.

A) Histogram showing the frequency of WT and mutation sequences in the bulk cell population transformed with each sgRNA construct. The horizontal axis indicates the number of colonies sequenced.

B) Automated electrophoresis of PCR amplified products using HLA-A outer/inner and HLA-B outer/inner specific PCR primer sets. Lanes #1-41 were examined genome-edited iPSC clones. Lane M, 10kb DNA size marker. Red arrows show the clones that were different size from WT or for which PCR products were not obtained.

C) Sequence alignment of Sanger reads of HLA-A from origin (WT) and Ff-I14s04 ABII KO #19. cyan: HLA-A sequence, green: PAM, black: HLA-A/HLA-B common sequence, yellow: HLA-B sequence.

D) Electropherograms of Sanger sequences at the target sites in genome-edited iPSC clones.

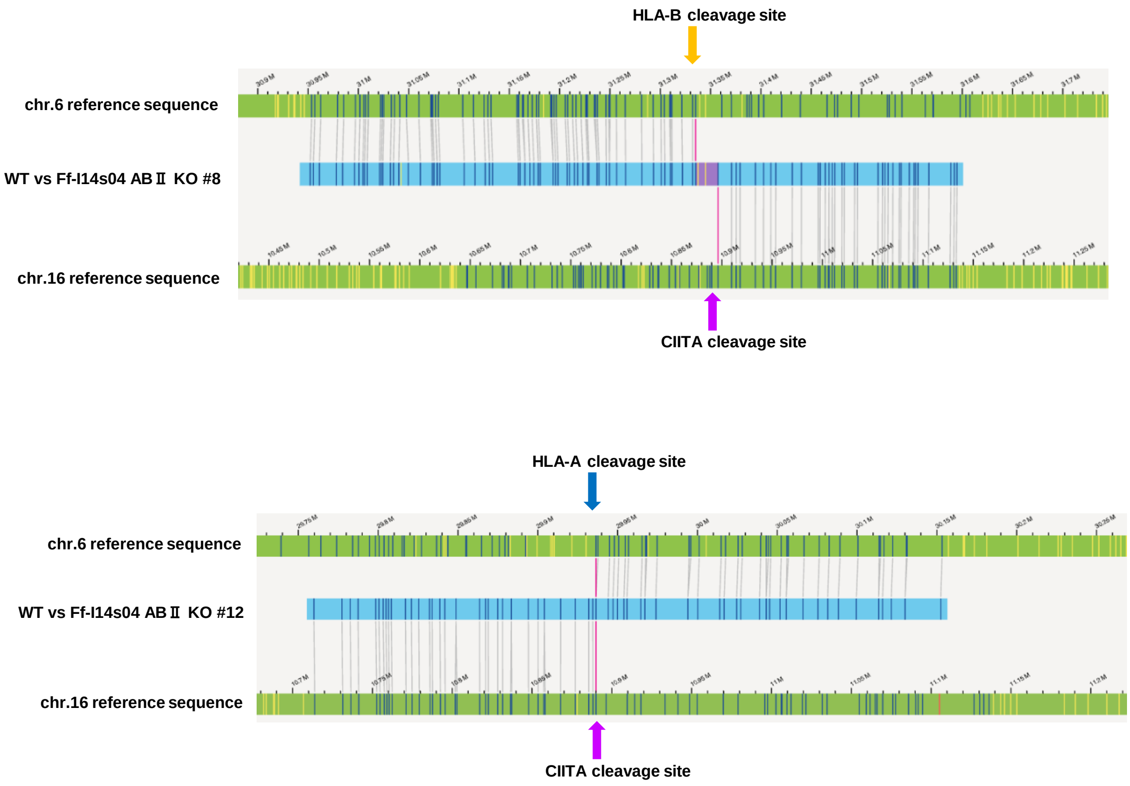

Figure S2. Complicated genome sequence shown by whole genome analysis.
The chromosomal translocations between HLA-A or HLA-B and CIITA cleavage site observed in optical genome mapping.
The genomic coordinate is hg38.

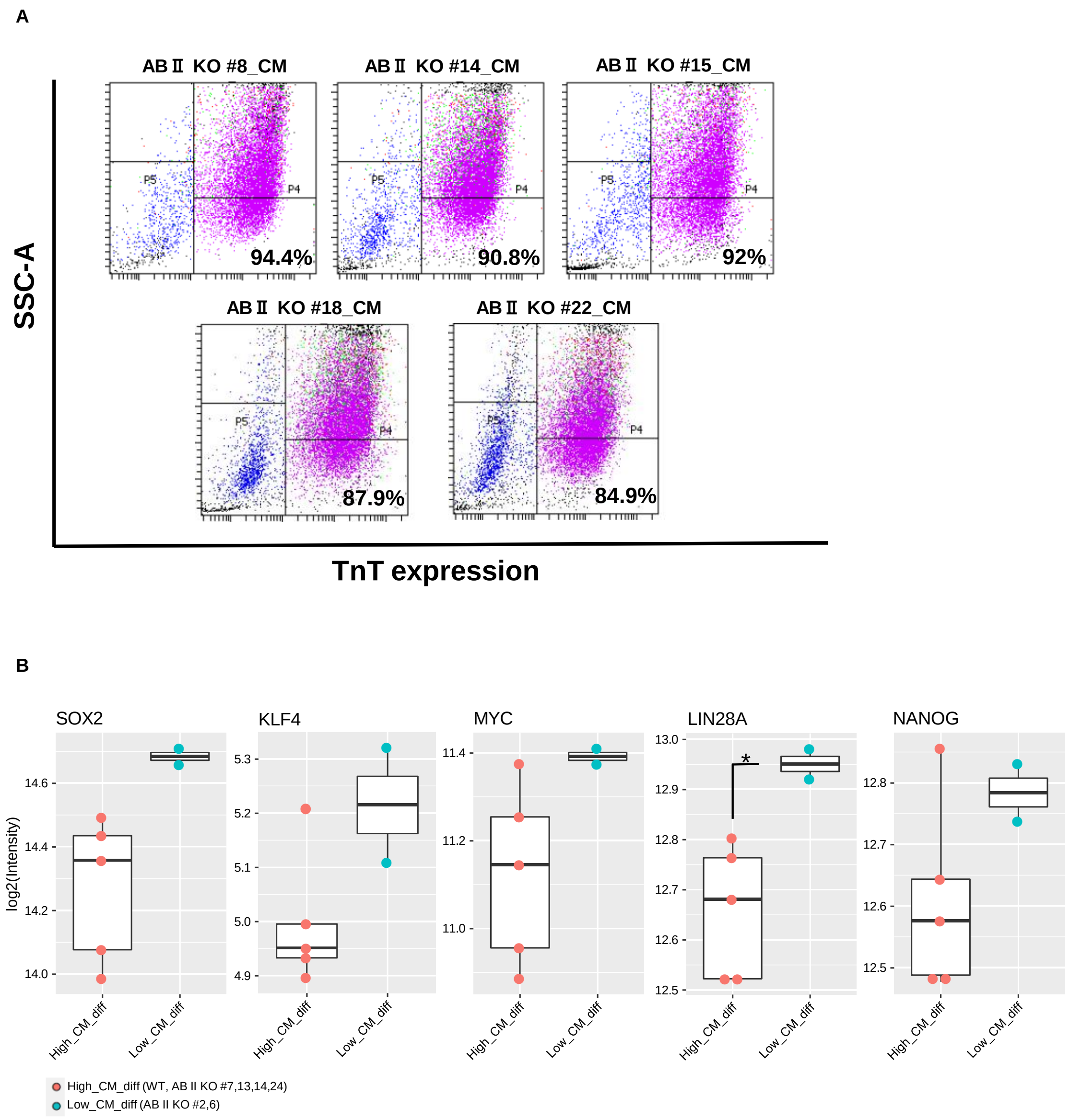

Figure S3. Evaluation of the cardiomyocyte differentiation potential of cells derived from genome-edited iPSCs.

A) Scatter plots of results of the FCM analysis examining the proportion of troponin T (TnT)+ cells after the induction of differentiation. The horizontal axis indicates the fluorescence intensity of fluorophores conjugated with the TnT antibody. The vertical axis indicates side scatter.

B) Boxplot of microarray data showing the expressions levels of pluripotent marker genes in iPSC clones showing high(orange) or low(cyan) cardiomyocyte differentiation potential. Each dot shows the clones. Asterisk indicates statistical significance: * $p < 0.05$.

A

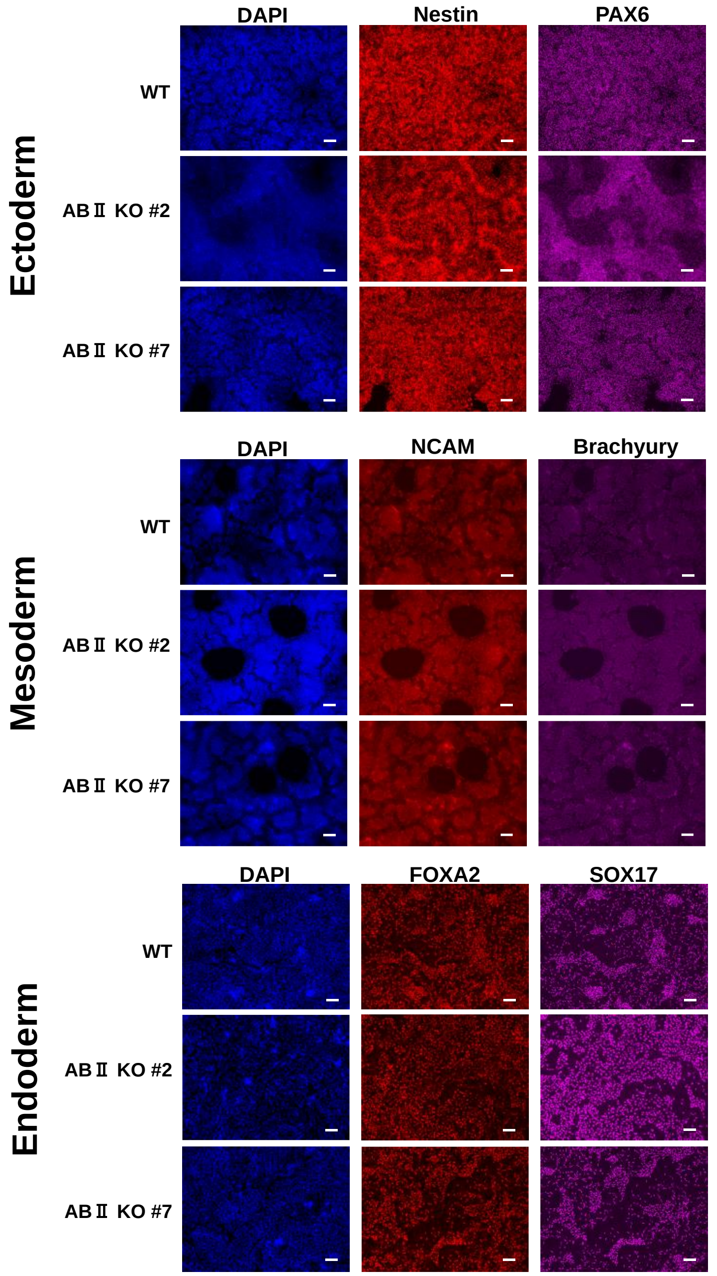

DAPI
Nestin
PAX6
Ectoderm
WT
ABⅡ KO #2
ABⅡ KO #7
DAPI
NCAM
Brachyury
Mesoderm
WT
ABⅡ KO #2
ABⅡ KO #7
DAPI
FOXA2
SOX17
Endoderm
WT
ABⅡ KO #2
ABⅡ KO #7

B

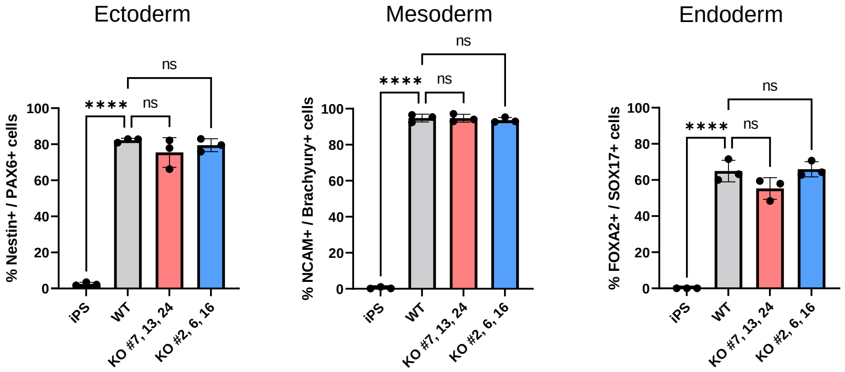

Ectoderm
% Nestin+ / PAX6+ cells
0
20
40
60
80
100
****
ns
ns
iPS
WT
KO #7, 13, 24
KO #2, 6, 16
Mesoderm
% NCAM+ / Brachyury+ cells
0
20
40
60
80
100
****
ns
ns
iPS
WT
KO #7, 13, 24
KO #2, 6, 16
Endoderm
% FOXA2+ / SOX17+ cells
0
20
40
60
80
100
****
ns
ns
iPS
WT
KO #7, 13, 24
KO #2, 6, 16

Figure S4. Capacity of differentiation into three germ layers.

A) Immunofluorescence micrographs of differentiation into three germ layers. Ectodermal differentiated cells were stained using Nestin and PAX6 antibodies (top). Mesodermal differentiated cells were stained using NCAM and Brachyury antibodies (middle). Endodermal differentiated cells were stained using FOXA2 and SOX17 antibodies (bottom). All cells were stained by DAPI for nuclear staining. Scale bar, 100 µm.

B) Quantification of three germ layer marker-positive cells by flow cytometry. ns, not significant. Asterisks indicate statistical significance: **** $p < 0.0001$.

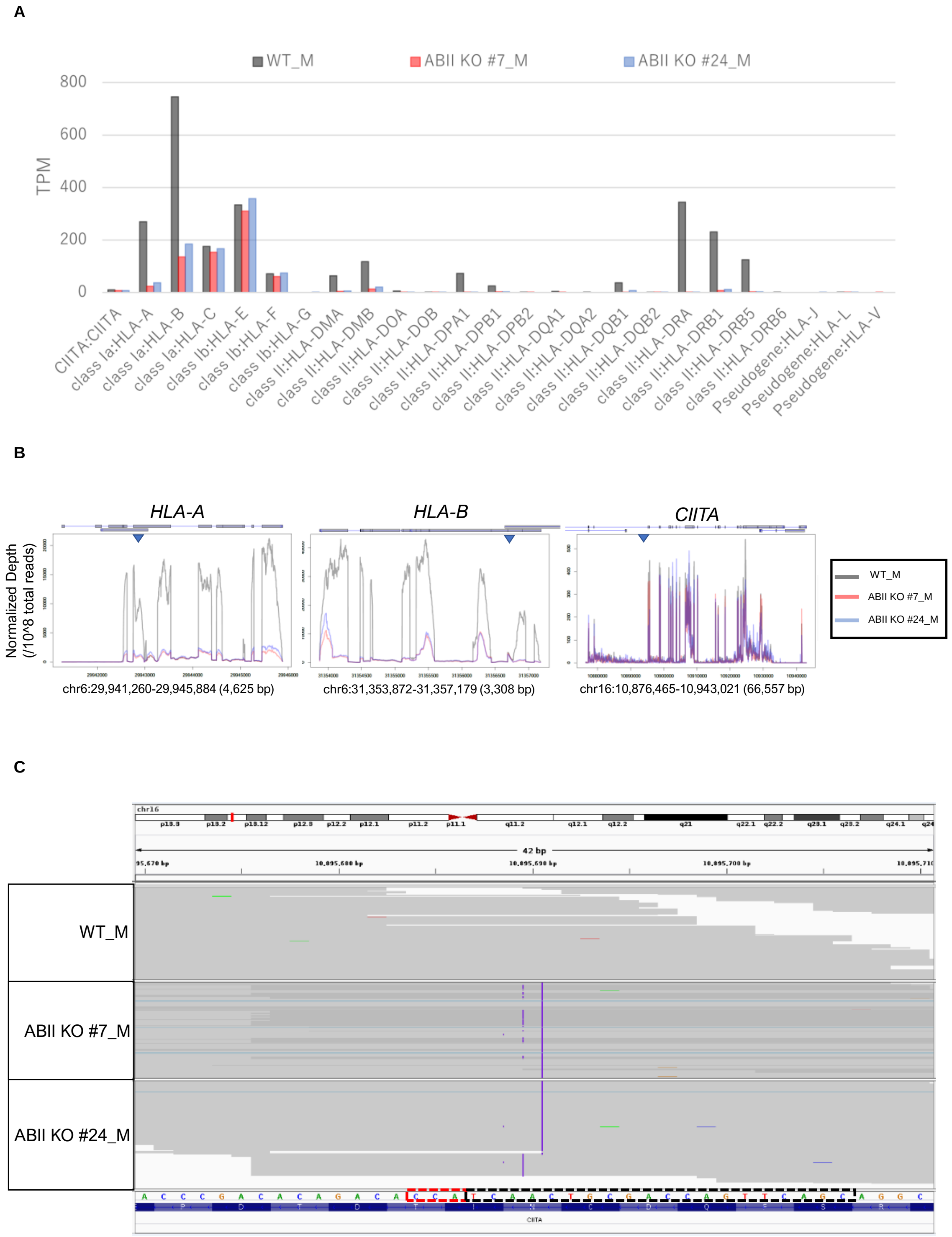

Figure S5. The mRNA expression of HLA-related genes in differentiated CD14+ monocytes derived from genome-edited iPSCs.

A) Transcripts per million (TPM) values showing the normalized gene expression of the HLA-related genes in CD14+ monocytes derived from genome-edited iPSC clones. CD14+ monocytes derived from WT and genome-edited #7 and #24 iPSCs are colored black, red and blue, respectively.

B) Diagram showing the normalized depth of the HLA-A, HLA-B and CIITA loci in differentiated monocytes derived from control and genome-edited iPSC clones (#7 and #24).

C) Integrated genome viewer (IGV) image showing the insertion of the base (purple vertical lines) in the sequence reads in ABII KO #7_M and ABII KO #24_M, which was not found in WT_M.

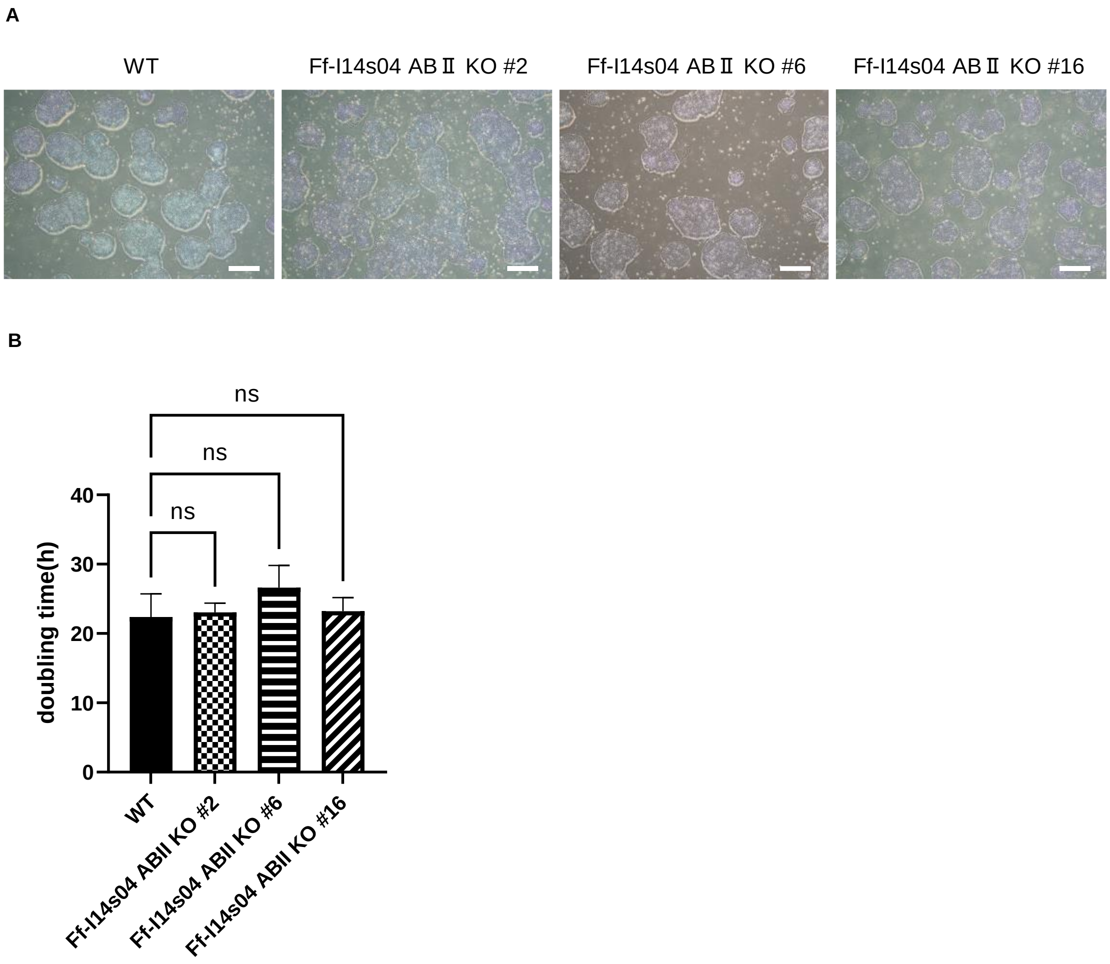

Figure S6. Differences in cell characteristics before and after genome editing.

A) Morphology of wild-type iPSC and HLA-A/B/CIITA genome-edited iPSC clones. Scale bar, 200 µm.

B) Barplot showing the doubling time of wild-type iPSC and genome-edited iPSC clones #2, #6 and #16. Three biological replicates were performed. ns, not significant.

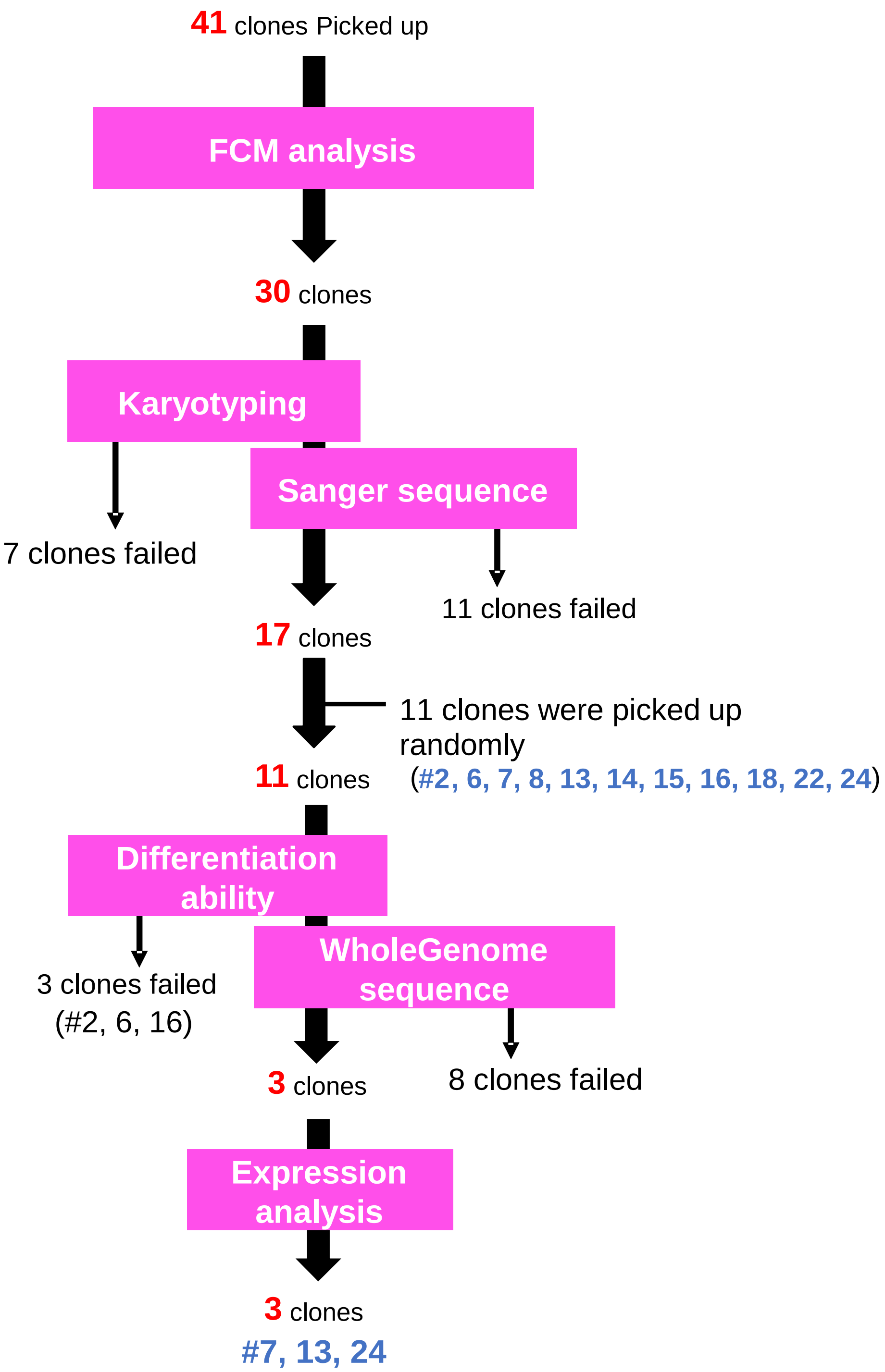

Figure S7. Summary of genome-edited iPSC clones selection.
We selected 30 clones with HLA-A$^{(-)}$/HLA-C$^{(+)}$ by FCM. We then examined the karyotype to assess genomic integrity and conducted Sanger sequencing to confirm the In/del pattern in the target regions. Thirteen clones failed this screening. Eleven clones were randomly selected for whole genome sequencing and a differentiation test. Three clones passed these tests. Finally, we assessed the gene expression level and obtained three genome-edited iPSC clones.

Table S1. HLA-A/B editable single guideRNA design.

Target gene : HLA-A and HLA-B

Target exon : exon2 or 3

PAM : NGG

Design tool : CRISPOR 4.99

Frequency ranking in Japanese population	Code of HLA-homozygous donors	HLA haplotype	Target exon	Sequences(5'-3')
1	QHJI	A*24:02<br>B*52:01	Exon 2	AGAGAACCTGCGGATCGCGC
2	RWMH	A*33:03<br>B*44:03	Exon 3	CAGGACCGCCTACGACGGCA
3	DRXT	A*24:02<br>B*07:02	Exon2	CCGTGTCCCGGCCCGGCCGC<br>GGGCTCCCCGCGGCCGGGCC<br>GCGGGCGCCGTGGATAGAGC<br>GGCCCCTCCTGCTCTATCCA
4	RJWI	A*24:02<br>B*54:01	Exon2	CGATGAAGCGGGGCTCCCCG

Table S2. Mutation patterns of target site-cloning and sequencing.

HLA-A	HLA-B	CIITA
del(deletion)-GA-re(reverse)	del-GAGAGAACC-for(forward)	del 100bp<-for
WT	ins-TCCTGCGACTTACCGC-for	del-CCTCCCAGAACCCGACACAGACACCATCAACTGCGACCAGT-for
del-CGAGA-re	WT	WT
del-CGAGA-re	del-GAACCTGC-rev	ins-A-for
del-GAGAGAACC-re	del-GA-for	del-CCATCAAC-rev
unclear	del-GA-rev	unclear
del-GAGAGAACC-re	WT	ins-A-rev
del-ACCTGCGGATCGCGCTCCGCTA-re	del-GAACCTGC-for	ins-A-for
ins(insertion)-A-re	WT	del-TGCGAC-rev
del-ACCTGCGGATCGCGCTCCGCTA-re	del-GAGAGAACC-rev	del-TCAACTGCGACCA-for
WT	del-AGAACCTG-rev	WT
		ins-AA-rev

Table S3. Amino acid predictions of the genome-edited iPSC clones.

Ff-I14s04 ABII KO	HLA-A		HLA-B		CIITA	
	Allele1	Allele2	Allele1	Allele2	Allele1	Allele2
#2	c.296_304del<br>p. Arg99_Asn101del		c.300_301delGA<br>p. Asn101Profs*37 Exon3		c.220_221insA<br>p. Asn74Lysfs*11 Exon3	c.220_221insC<br>p. Asn74Thrfs*11 Exon3
#6	c.300_311del<br>p. Glu100Asp / p. Asn101_Ile104del		c.294_299del<br>p. Try98* Exon2		c.220_221insA<br>p. Asn74Lysfs*11 Exon3	
#7	c.299_300insA<br>p. Asn101Glufs*120 Exon4	c.299delA<br>p. Glu100Glyfs*22 Exone2	c.299_300insT<br>p. Glu100Aspfs*39 Exon3	c.300_306del<br>p. Glu100Aspfs*49 Exon3	c.220_221insA<br>p. Asn74Lysfs*11 Exon3	
#8	c.299_300insC<br>p. Glu100Aspfs*121 Exon4		c.300_301delGA<br>p. Asn101Profs*37 Exon3		c.220_221insA<br>p. Asn74Lysfs*11 Exon3	
#13	c.299_300insA<br>p. Asn101Glufs*120 Exon4		c.299_300insA<br>p. Asn101Glufs*38 Exon3		c.220_221insA<br>p. Asn74Lysfs*11 Exon3	
#14	c.296_304del<br>p. Arg99_Asn101del		c.300_301delGA<br>p. Asn101Profs*37 Exon3	c.289_314del<br>p. Thr97Alafs*33 Exon3	c.220_221insA<br>p. Asn74Lysfs*11 Exon3	
#15	c.283_299del<br>p. Ser95Glufs*120 Exon4		c.299_300insG<br>p. Asn101Glufs*38 Exon3		c.220_221insA<br>p. Asn74Lysfs*11 Exon3	c.220_221insT<br>p. Asn74Ilefs*11 Exon3
#16	c.299_300insA<br>p. Asn101Glufs*120 Exon4		c.299_300insC<br>p. Glu100Aspfs*39 Exon3		c.220_221insA<br>p. Asn74Lysfs*11 Exon3	c.221_228del<br>p. Asn74Thrfs*8 Exon3
#18	c.284_299del<br>p. Ser95* Exon2		c.299_300insA<br>p. Asn101Glufs*38 Exon3		c.220_221insC<br>p. Asn74Thrfs*11 Exon3	c.212_231del<br>p. Asp71Valfs*7 Exon3
#22	c.300_301delGA<br>p. Asn101Profs*119 Exon4		c.300_302delGAA<br>p. Glu100* / p. Asn101Asp		c.220_221insA<br>p. Asn74Lysfs*11 Exon3	c.220_221insAA<br>p. Asn74Lysfs*42 Exon4
#24	c.300delG<br>p. Asn101Thrfs*21 Exon2	c.300_310del<br>p. Glu100Aspfs*117 Exone4	c.299_300insA<br>p. Asn101Glufs*38 Exon3	c.300_307del<br>p. Asn101Aspfs*35 Exon3	c.220_221insA<br>p. Asn74Lysfs*11 Exon3	

Table S4. Denovo CNV mutation in genome edited cells.

ABII_ KO_2	ABII_ KO_6	ABII_ KO_7	ABII_ KO_8	ABII_ KO_13	ABII_ KO_14	ABII_ KO_15	ABII_ KO_16	ABII_ KO_18	ABII_ KO_22	ABII_ KO_24	Locus	Size(bp)	Overlap_Gene	Census (v88)	Off-target
X											chr3:128,339,436-128,341,007	1572	RPN1	RPN1	CIITA
										X	chr4:145,837,936-145,841,174	3239	-	-	
X	X						X				chr6:29,911,740-31,327,897	1416158	HLA-A,HCG9,ZNRD1-AS1,HLA-J,HCG8,ZNRD1,PPP1R11,RNF39, TRIM31,TRIM31-AS1,TRIM40,TRIM10,TRIM15,TRIM26,HCG17, HLA-L,MIR6891,HCG18,TRIM39,TRIM39-RPP21,RPP21,HLA-E, GNL1,PRR3,ABCF1,MIR877,PPP1R10,MRPS18B,ATAT1,C6orf136, DHX16,PPP1R18,NRM,MDC1,MDC1-AS1,TUBB,FLOT1,IER3,LINC00243, DDR1,MIR4640,GTF2H4,VARS2,SFTA2,DPCR1,MUC21,MUC22,HCG22, C6orf15,PSORS1C1,CDSN,PSORS1C2,CCHCR1,TCF19,POU5F1,PSORS1C3, HCG27,HLA-C,HLA-B	HLA-A, POU5F1	
								X			chr9:71,733,413-71,752,214	18802	TJP2	-	
								X			chr9:104,715,123-104,726,036	10914	-	-	
								X			chr9:105,545,015-105,566,729	21715	-	-	
								X			chr9:138,918,206-138,937,605	19400	NACC2	-	
								X			chr9:140,975,937-140,988,910	12974	CACNA1B	-	
										X	chr16:10,988,440-10,989,661	1222	CIITA	CIITA	
							X				chr17:44,442,296-44,629,670	187375	NSFP1,ARL17A,LRRC37A2	-	
	X										chr19:5,819,865-5,862,124	42260	NRTN,FUT6,FUT3,LOC101928844	-	
					X						chr21:25,118,166-25,121,850	3685	-	-	

Table S5. Denovo SNV mutation in genome edited cells.

ABII_ KO_2	ABII_ KO_6	ABII_ KO_7	ABII_ KO_8	ABII_ KO_13	ABII_ KO_14	ABII_ KO_15	ABII_ KO_16	ABII_ KO_18	ABII_ KO_22	ABII_ KO_24	Chr	Start	End	Ref	Alt	Func.ref Gene	Gene.ref Gene	ExonicFunc .ref Gene
x	x	x	x	x	x	x	x		x	x	chr16	10,989,545	10,989,545	-	A	exonic	CIITA	fs[a] insertion
x								x			chr16	10,989,546	10,989,546	-	C	exonic	CIITA	fs insertion
x											chr1	24,676,608	24,676,608	C	G	exonic	GRHL3	ns[b] SNV
x											chr9	19,290,830	19,290,830	C	A	exonic	DENND4C	ns SNV
x					x						chr6	29,910,752	29,910,760	GACCGAGTG	-	exonic	HLA-A	non fs deletion
	x										chr1	143,767,518	143,767,518	G	T	exonic	PPIAL4G	ns SNV
	x										chr6	28,541,552	28,541,552	G	T	exonic	ZBED9	ns SNV
	x									x	chr6	29,910,758	29,910,768	GTGGACCTGGG	-	exonic	HLA-A	fs deletion
		x									chr12	32,949,161	32,949,161	T	A	exonic	PKP2	ns SNV
		x	x	x			x				chr6	29,910,758	29,910,758	-	A	exonic	HLA-A	fs insertion
		x									chr6	31,324,502	31,324,508	CAGGCTC	-	exonic	HLA-B	fs deletion
					x						chr8	101,178,090	101,178,090	G	T	exonic	SPAG1	ns SNV
					x						chr15	72,063,533	72,063,533	G	A	exonic	THSD4	ns SNV
					x						chr18	29,264,323	29,264,325	AGA	-	exonic	B4GALT6	non fs deletion
					x						chr6	31,324,506	31,324,507	CT	-	exonic	HLA-B	fs deletion
						x					chr12	278,268	278,268	A	T	exonic	IQSEC3	ns SNV
						x					chr16	10,989,546	10,989,546	-	T	exonic	CIITA	fs insertion
						x					chr19	39,200,939	39,200,939	C	T	exonic	ACTN4	ns SNV
							x				chr12	120,762,766	120,762,766	G	A	exonic	PLA2G1B	ns SNV
							x				chr16	10,989,548	10,989,555	CTGCGACC	-	exonic	CIITA	fs deletion
								x			chr2	172,966,269	172,966,269	C	T	exonic	DLX2	ns SNV
								x			chr3	11,059,013	11,059,013	C	A	exonic	SLC6A1	ns SNV

								x			chr3	48,696,834	48,696,834	C	A	exonic	CELSR3	ns SNV
								x			chr16	10,989,535	10,989,554	CAGACACCATCAACTGCGAC	-	exonic	CIITA	fs deletion
								x			chr21	46,945,842	46,945,842	C	G	exonic	SLC19A1	ns SNV
								x			chr6	29,910,754	29,910,761	CCGAGTGG	-	exonic	HLA-A	fs deletion
									x		chr3	40,464,893	40,464,893	G	A	splicing	ENTPD3	-
									x		chr12	7,288,877	7,288,877	G	C	exonic	CLSTN3	ns SNV
									x		chr16	10,989,545	10,989,545	-	AA	exonic	CIITA	fs insertion
									x		chr19	13,934,185	13,934,185	C	T	exonic	ZSWIM4	ns SNV
									x		chr4	88,535,868	88,535,868	A	G	exonic	DSPP	ns SNV
									x		chr6	29,910,758	29,910,759	GT	-	exonic	HLA-A	fs deletion
										x	chr4	73,003,798	73,003,798	G	C	exonic	NPFFR2	ns SNV
										x	chr12	6,675,434	6,675,434	C	T	exonic	NOP2	ns SNV
										x	chr6	31,324,500	31,324,507	CGCAGGCT	-	exonic	HLA-B	fs deletion

[a] fs means frameshift

[b] ns means non synonymous

Table S6. Primer Information.

PCR primer oligo name	PCR primer oligo sequence
1383D2-HLA-A-be-ex1-fwd	TTGGGGATTCCCCAACTCC
HLA-A-in7-rev	GTCCACTGTTCCGCCCAA
HLA-A24:02-ex2-fwd	CTCCCACTCCATGAGGTATTTCTC
HLA-A24:02-ex3-rev	CCCTCCAGGTAGGCTCTCT
1383D2-HLA-B-be-ex1-fwd	CAGGATACTCGTGACGCGT
1383D2-HLA-B-in7-rev	ACACACGCGAAACATCCCAA
B52-ex2-fwd	CATGAGGTATTTCTACACCGCCA
0706HLA-B-in-rev	CTGGTACCCGCGCGCTG
604B1-HLA-C-be-ex1-fwd	CAATCAGCGTCTCCGCAGT
604B1-HLA-C-ex8-rev	ATGCTAACAGGAACGCAGACA
HLA-C-ex1-fwd	TCCTGCTGCTCTCGGGAG
CIITA-Fwd	GCTTGCTGTAGAGACGGCAAT
CIITA-Rev	TTGCTCCGCATCCCCCTT
M13 forward primer	GTAAAACGACGGCCAG
M13 reverse primer	CAGGAAACAGCTATGAC

Data S1. On/off-target candidate of HLA-A/B guideRNA.

Data S2. On/off-target candidate of CIITA guideRNA.

Data S3. Statistical analysis.
